# Excitation-mode-selective emission through multiexcitonic states in a double perovskite single crystal

**DOI:** 10.1038/s41377-024-01689-7

**Published:** 2025-01-02

**Authors:** Hao Suo, Nan Wang, Yu Zhang, Xin Zhang, Jinmeng Xiang, Xiaojia Wang, Guansheng Xing, Dongxu Guo, Jiwen Chang, Yu Wang, Panlai Li, Zhijun Wang, Yuhai Zhang, Bing Chen, Shuzhou Li, Chongfeng Guo, Feng Wang

**Affiliations:** 1 Hebei Key Laboratory of Optic-electronic Information and Materials, College of Physics Science & Technology, Hebei University, Baoding, 071002 China; 2 Department of Materials Science and Engineering, City University of Hong Kong, Kowloon, 999077 Hong Kong SAR China; 3 State Key Laboratory of Photon-Technology in Western China Energy, Institute of Photonics & Photon-Technology, Northwest University, Xi’an, 710127 China; 4 Institute for Advanced Interdisciplinary Research, University of Jinan, Jinan, Shandong 250022 China; 5 College of Electronic and Optical Engineering & College of Flexible Electronics (Future Technology), Nanjing University of Posts and Telecommunications, Nanjing, 210023 China; 6 School of Materials Science and Engineering, Nanyang Technological University, 50 Nanyang Avenue, Singapore, 639798 Singapore

**Keywords:** Optical materials and structures, Inorganic LEDs

## Abstract

Low-dimensional lead-free metal halide perovskites are highly attractive for cutting-edge optoelectronic applications. Herein, we report a class of scandium-based double perovskite crystals comprising antimony dopants that can generate multiexcitonic emissions in the ultraviolet, blue, and yellow spectral regions. Owing to the zero-dimensional nature of the crystal lattice that minimizes energy crosstalk, different excitonic states in the crystals can be selectively excited by ultraviolet light, X-ray irradiation, and mechanical action, enabling dynamic control of steady/transient-state spectral features by modulating the excitation modes. Remarkably, the transparent crystal exhibits highly efficient white photoluminescence (quantum yield >97%), X-ray excited blue emission with long afterglow (duration >9 h), and high-brightness self-reproducible violet-blue mechanoluminescence. These findings reveal the exceptional capability of low-dimensional perovskite crystals for integrating various excitonic luminescence, offering exciting opportunities for multi-level data encryption and all-in-one authentication technologies.

## Abstract (web summary)

Highly variable STE luminescence is realized within a transparent crystal composed of antimony-doped double perovskite. By controlling excitation conditions, we achieve highly efficient white photoluminescence (quantum yield >97%), X-ray excited blue emission with long afterglow (duration >9 h), and self-reproducible violet-blue mechanoluminescence. We show that the excitation-mode-selective emission can be harnessed for multi-level data encryption and authentication technologies.

## Graphical abstract

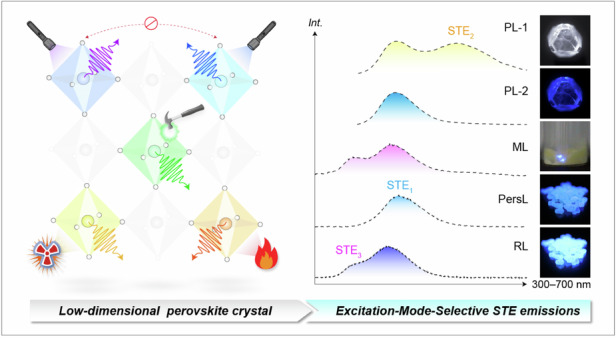

## Introduction

**Fig. 1:**
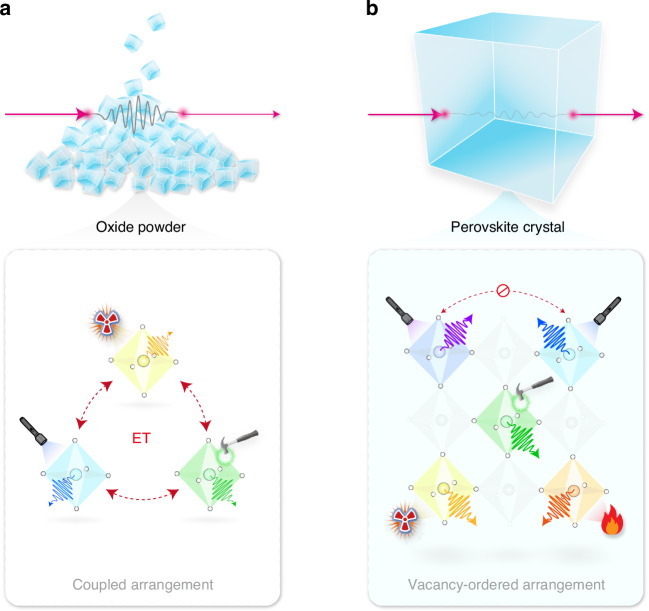
Schematic illustration of excitation-mode-selective emissions **a** Reported oxide powders with complicated dopant compositions prepared by high-temperature synthesis. These material systems with the coupled arrangement of dopant polyhedrons typically feature low efficiency and imbalanced performance due to unwanted energy transfer (ET) between dopants and severe random scattering. **b** Proposed low-dimensional metal halide perovskite crystal with energetically decoupled multiexcitonic states. The transparent crystal features minimal energy exchange interactions between STEs and reduced light scattering, favoring selective STE activation with improved luminescence efficiency and output by a given excitation

Smart luminescent crystals that show switchable light emissions in response to different forms of excitations have attracted pervasive attention in a wide array of forefront applications such as sensing, optoelectronics, anti-counterfeiting, and data storage^1–5^. In particular, the flexibility and diversity afforded by ion luminescence endow impurity-doped inorganic luminescent materials with enormous superiorities in realizing excitation-selective luminescence^6^. For example, the dynamical control of optical features by leveraging multiple excitation modes, such as ultraviolet (UV) and near-infrared (NIR) photons, X-ray irradiation, and mechanical force, has been explored in various oxide matrixes co- or tri-doped with lanthanide or transition metal ions^7–10^. However, these powder systems typically suffer from low efficiency and imbalanced performance due to unwanted crosstalk between dopants and severe random scattering (Fig. 1a). In addition, these oxide compounds are often reliant on high-temperature synthesis, setting formidable obstacles in their practical applications^11,12^.

As an alternative to ion luminescence, self-trapped exciton (STE) in low-dimensional metal halide perovskite crystals has been proven useful for generating excitation-mode-selective luminescence due to its broadband spectrum, negligible self-absorption, and high photoluminescence quantum yield (PLQY)^13–21^. The vacancy-ordered arrangement of low-dimensional crystals results in mutually independent STEs with minimal energy exchange interactions, which is favorable for selective STE activation by a given excitation^22^. Compared to agglomerated powders, the transparent nature of perovskite single crystals effectively reduces the scattering effect, thereby rendering an improved photon transmission and outputs (Fig. 1b)^23,24^. For example, the deliberated control of photon-excitation wavelengths has enabled dynamic switching of multiexcitonic emissions in several zero-dimensional halide perovskites^25,26^. Despite the success in achieving tunable photoluminescence (PL), extended control over STE emission behaviors using X-ray and mechanical excitations has not been established in these perovskite crystals. Particularly, mechanically excited luminescence, known as mechanoluminescence (ML), is mostly observed in material systems synthesized under high temperatures^27–30^, which remains inaccessible to STEs in calcination-free inorganic perovskite crystals. In this regard, low-dimensional metal halide perovskite crystals with enhanced optical capabilities under multiple excitation modes are in high demand.

In this work, we report highly variable and multimodally excitable STE luminescence within a single component of all-inorganic halide double perovskite crystal doped with antimony ions. We identify three mutually independent STEs related to the host and dopant that can be selectively populated through different excitation pathways. Accordingly, we achieve chromatic and temporal tuning of highly efficient multiexcitonic emissions by controlling excitation conditions. Significantly, we realize temporally resolvable STE emissions with distinct time decay behaviors under X-ray irradiation and mechanical action for the first time. We show that the excitation-mode-selective emission can be harnessed for multi-level data encryption and authentication technologies.

## Results

### Synthesis and structural characterization

**Fig. 2:**
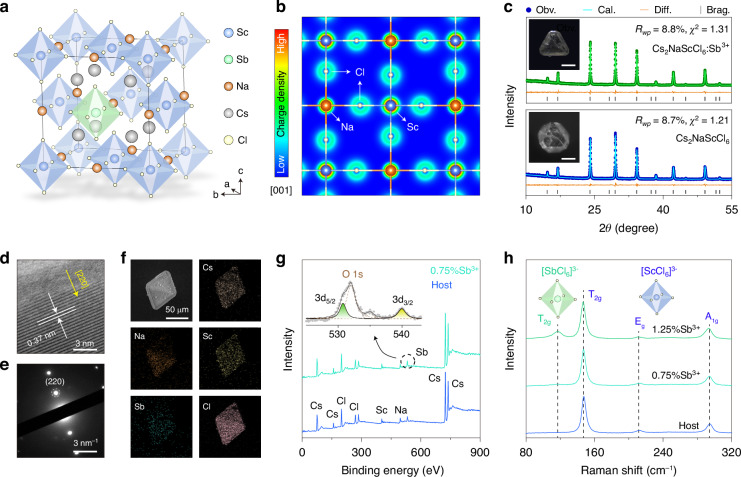
Structural characterizations of Cs_2_NaScCl_6_:Sb^3+^ crystals **a** Schematic illustration of Cs_2_NaScCl_6_ crystal structure highlighting [ScCl_6_]^3−^ and [SbCl_6_]^3−^ octahedrons. **b** Charge density isosurface at (001) facet in Cs_2_NaScCl_6_ showing the decoupling of electronic orbitals between neighboring [ScCl_6_]^3−^ units. **c** Rietveld refinement of XRD patterns for Cs_2_NaScCl_6_ and Cs_2_NaScCl_6_:Sb^3+^ (0.75%) crystals. Insets show the photographs of millimeter-sized single crystals under daylight conditions. Scale bar = 1 mm. **d** HR-TEM image, **e** SAED pattern, and **f** element mapping of a randomly selected small microcrystal. **g** XPS survey of Cs_2_NaScCl_6_ and Cs_2_NaScCl_6_:Sb^3+^ crystals. Inset shows the high-resolution spectrum of Sb 3 d. Note that the two peaks at 531.8 and 535 eV were assigned to 1 s of O^2−^. **h** Raman spectra of Cs_2_NaScCl_6_ and Cs_2_NaScCl_6_:Sb^3+^ (0.75%, 1.25%) crystals

Our study employed all-inorganic rare-earth halide double perovskite host Cs_2_NaScCl_6_ due to its high defect tolerances, excellent stability, and high doping capacity^31,32^. Cs_2_NaScCl_6_ crystalizes in a rock-salt face-centered cubic structure (space group: *Fm-3m*), where the corner-sharing [NaCl_6_]^5−^ and [ScCl_6_]^3−^ octahedral units are alternately arranged to form the highly ordered three-dimensional (3D) framework (Fig. 2a). This arrangement effectively reduces the electronic dimensionality by decoupling electronic orbitals of nearest [ScCl_6_]^3−^ by [NaCl_6_]^5−^ octahedrons, favoring STE formation (Fig. 2b). Trivalent antimony (Sb^3+^) ion with an *ns*^*2*^ electronic configuration was selected to introduce effective STE emissions by forming Sb^3+^-based polyhedrons. In theory, the Sb^3+^ ion (*r* = 0.76 Å) is expected to occupy the octahedral Sc^3+^ site (*r* = 0.745 Å) with *O*_*h*_ point symmetry due to the close ionic radii and the same valence state. This site occupation scheme is supported by the density functional theory (DFT) calculations, which disclosed a lower formation energy (*E*_*form*_) of Sb^3+^ in the Sc^3+^ site than in the Na^+^ or Cs^+^ site (Table S1).

Through a modified hydrothermal synthesis, millimeter-sized Sb^3+^-doped Cs_2_NaScCl_6_ single crystals were grown from concentrated hydrochloric acid via a cooling-induced crystallization. The resulting colorless transparent crystals were confirmed to be a pure cubic elpasolite structure with high crystallinity by powder X-ray diffraction (XRD, Fig. 2c). Rietveld refinement analysis revealed a gradual increase in the volume of the unit cell and [ScCl_6_]^3−^ octahedron as the Sb^3+^ doping concentration increased, validating the successful substitution of bigger Sb^3+^ for Sc^3+^ (Fig. S1 and Table S2). In addition, the high-resolution transmission electron microscopy (HR-TEM) image and selected area electron diffraction (SAED) pattern revealed the single-crystalline nature of the as-prepared crystals with high crystallinity (Fig. 2d, e).

Compositional analysis by energy dispersive X-ray (EDX) spectroscopy indicated the homogeneous distribution of the host and dopant elements within a single microcrystal (Fig. 2f). The successful incorporation of Sb^3+^ was further supported by X-ray photoelectron spectroscopy (XPS), where the binding energy information of constituent elements in Cs_2_NaScCl_6_ was clearly detected (Fig. 2g). After doping with antimony ions, the appearance of two additional peaks at 530.7 and 540.0 eV, corresponding to 3d_5/2_ and 3d_3/2_ of Sb^3+^, demonstrated the existence of trivalent antimony without a secondary valence state. Furthermore, the lattice vibration information related to the cation-based polyhedron was examined by Raman spectroscopy (Fig. 2h). In specific, three sharp peaks at 146.9, 221.6, and 294 cm^−1^ were clearly observed in bare (undoped) Cs_2_NaScCl_6_ crystal, which can be assigned to T_2g_, E_g_, and A_1g_ vibration modes of [ScCl_6_]^3−^ octahedron. Notably, the introduction of Sb^3+^ ions led to the emergence of a new band at 116.2 cm^−1^ corresponding to the T_2g_ bending mode of [SbCl_6_]^3−^ unit, further proving the occupation of Sb^3+^ at the Sc^3+^ site^33^.

### UV light-excited multiexcitonic emissions

**Fig. 3:**
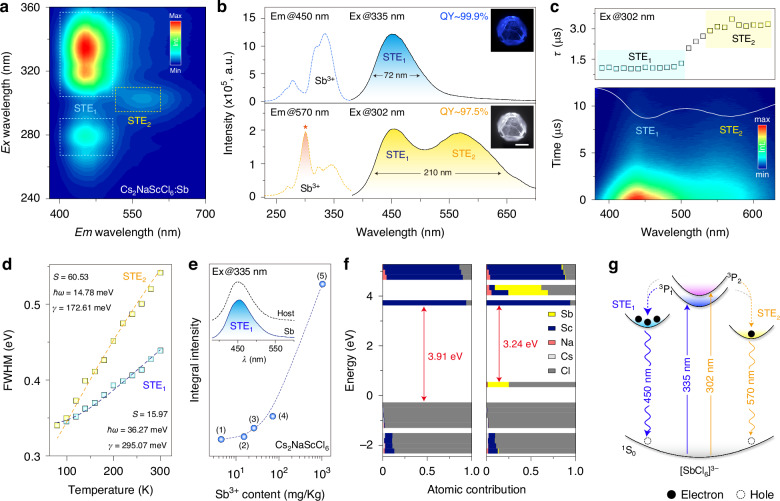
UV light-excited multiexcitonic emissions **a** Contour plot of the excitation-wavelength-resolved PL spectra of Cs_2_NaScCl_6_:Sb^3+^ (0.75%) crystal highlighting its dual-excitonic emissions. Note that the blue emission dominated over the yellow one except under 302 nm excitation. **b** PLE (dotted lines) and PL (solid lines) spectra of Cs_2_NaScCl_6_:Sb^3+^crystal, and insets show the optical images of single crystals under UV excitation at 302 and 335 nm. Scale bar = 1 mm. **c** Time-resolved PL mapping and steady-state PL spectrum under 302 nm excitation, along with the calculated average lifetimes versus the emission wavelengths. **d** The plot of FWHM of blue and yellow emissions versus the temperature. By fitting the experimental data, the parameters were derived to ℏ*ω*_*op*_ = 36.27 meV, *S* = 15.97, *γ*_op_ = 295.07 meV for blue emission and ℏ*ω*_*op*_ = 14.78 meV, *S* = 60.53, *γ*_op_ = 172.61 meV for yellow one, respectively. **e** The integral intensity of Cs_2_NaScCl_6_ and Cs_2_NaScCl_6_:Sb^3+^ (0.02%) crystals under 335 nm excitation as a function of Sb^3+^ content detected by ICP-MS analysis, and the insets show the corresponding PL spectra. Note that (1–4) represented bare Cs_2_NaScCl_6_ prepared using ScCl_3_·6H_2_O-Aladdin, ScC_6_H_9_O_6_·xH_2_O-Aladdin, Sc_2_O_3_-Aladdin, or Sc_2_O_3_-Alfa Aesar, while (5) represented Cs_2_NaScCl_6_:Sb^3+^ (0.02%) crystal. **f** The calculated electronic structure of bare and Sb^3+^-doped Cs_2_NaScCl_6_ crystals. **g** The schematic energy level diagram showing the excitation and STE emission processes

We next investigated the photoluminescence (PL) properties of Sb^3+^-doped Cs_2_NaScCl_6_ crystals. Absorption spectroscopy revealed intense absorptions in the range of 290–310 nm and 320–450 nm, which might be assigned to the ^1^S_0_→^3^P_1_ and ^3^P_2_ transitions of Sb^3+^, respectively (Fig. S2). Accordingly, the PL intensity map was constructed against the excitation in the relevant wavelength range, in which two distinct emission bands were detected in the blue and yellow spectral regions (Fig. 3a). By excitation into ^1^S_0_→^3^P_1_ at 335 nm, Cs_2_NaScCl_6_:Sb^3+^ crystal exhibited an intense broad emission band centered at 450 nm with a full width at half maximum (FWHM) of 72 nm (Fig. 3b). In contrast, dual emissions at 450 and 570 nm were observed under 302 nm excitation, registering an intense white emission with an ultra-large FWHM of 210 nm. The corresponding excitation process might be attributed to the ^1^S_0_→^3^P_2_ transition^34,35^. Notably, the PL switching from blue to white emission was all detectable in the samples doped with different Sb^3+^ contents by changing the excitation wavelength from 335 to 302 nm (Fig. S3). This excitation-wavelength-controlled PL switching behavior is essentially independent of Sb doping concentration (Fig. S4). By correlating PLQY with the doping level, we determined an optimal Sb^3+^ concentration of 0.75% with near-unity PLQY values for blue (~ 99.9%) and white (~97.5%) emissions, outperforming the reported values in most of luminescence materials (Figs. S5–S6 and Table S3).

Time-resolved PL spectra recorded clearly different decay kinetics between the blue and yellow emissions, which were well fitted by a bi-exponential function, further demonstrating the existence of two Sb-related luminescent centers (Fig. 3c and Fig. S7). Note that the evolution of average lifetimes in the 500–540 nm range was ascribed to the gradual domination of the slow-decay process (*τ*_yellow_) over the fast-decay counterpart (*τ*_blue_). The lifetimes of blue and yellow emissions, which were nearly unaffected by the dopant concentration, were calculated to be 1.08 and 3.44 μ*s*, respectively. In addition, the lifetime at 450 nm hardly varied as the switching excitation wavelength from 335 to 302 nm, indicating the negligible energy exchange between the two PL components due to the spatially separated polyhedral units (Fig. S8). The microsecond-scale decay behaviors can rule out the origin of the broadband emissions from the free exciton (FE) transition. In addition, we found a perfect linear dependence of PL intensity on the excitation power density without saturation, thereby excluding the contribution of lattice defect states to the broadband emission (Fig. S9).

We thus deduced that the broadband emissions stem from the STE recombination, relevant to the Jahn-Teller distortion of octahedrons^22^. In this case, the strength of electron-phonon coupling strongly determines the formation tendency of STE emission, which can be evaluated by Huang-Rhys factor (*S*) and Fröhlich coupling constant (*γ*_op_) using the following equations^36^:

1 $${\rm{FWHM}}(T)=2.36\sqrt{{\rm{S}}}\hslash {\omega }_{{\rm{op}}}\sqrt{\coth \frac{\hslash {\omega }_{{\rm{op}}}}{{{\rm{2k}}}_{{\rm{B}}}{\rm{T}}}}$$

2 $$\varGamma (T)={\Gamma }_{0}+{\gamma }_{{\rm{op}}}{({{\rm{e}}}^{\hslash {\omega }_{{\rm{op}}}/{{\rm{k}}}_{{\rm{B}}}{\rm{T}}}-1)}^{-1}$$

where $\hslash$*ω*_op_, *k*_*B*_, and *T* denote the longitudinal optical phonon energy, Boltzmann constant, and temperature, respectively. ${\varGamma }_{0}$ is a temperature-independent term related to the disorder and imperfections of the lattice, and the second term of Eq. (2) represents the homogeneous broadening induced by scattering from acoustic and longitudinal optical phonons. Accordingly, these parameters were derived by fitting the temperature-dependent emission bandwidth (Fig. 3d and Fig. S10). The large *S* values unveiled the strong electron-phonon interactions in Cs_2_NaScCl_6_:Sb^3+^ crystal, which facilitated the generation of efficient STE emissions by inducing intensive elastic distortions of the excited state lattice.

Previously, the high-energy blue emission of rare-earth halide double-perovskites was primarily ascribed to the intrinsic host STE recombination, which can be substantially brightened upon Sb^3+^ doping^31,37,38^. In line with previous studies, we detected broadband emissions in bare Cs_2_NaRECl_6_ (RE = Sc, Lu, Y, and Gd) crystals that showed a close spectral resemblance to that of Sb^3+^-doped counterparts except for the lower PLQYs (Figs. S11–13). However, our investigation indicates that the high-energy STE emission is more likely attributed to the Sb^3+^ ion rather than the perovskite host, as we detected a trace amount of Sb element in bare crystals by ICP-MS analysis (Table S4). The nearly identical transient and steady-state spectroscopic properties also suggest the consistent origin of the broadband emission before and after doping with Sb^3+^ ions (Fig. S14). Due to the difficulties of separating Sb impurity from scandium sources, the STE emission was consistently detectable under replicated synthesis using reagents purchased from different suppliers (Fig. S15). On top of that, the emission intensity of the as-prepared crystals was positively correlated with the detected Sb^3+^ content, further supporting that the high-energy broadband emission stems from the STE recombination in [SbCl_6_]^3−^ unit (Fig. 3e).

As the yellow emission was straightforwardly detected by introducing a trace amount of Sb^3+^ (0.02%), it is unlikely ascribed to the doping-induced host STE states or impurity phases of Cs_2_ScCl_5_·H_2_O and Cs_3_Sb_2_Cl_9_ (Figs. S16–17). Notably, a similar yellow emission was also detected in Cs_2_NaRECl_6_:Sb^3+^ crystals, yet the intensity was extremely weak even at 4 K (Figs. S18–19). Moreover, this yellow emission was gradually attenuated at RT by replacing Sc^3+^ with other RE^3+^ ions (Fig. S20). We thus infer that the Sc-based crystal lattice plays a vital role in the low-energy STE emission at 570 nm. The smallest ionic radius of Sc^3+^ across the rare-earth series may allow a relatively distorted lattice environment, which is essential for the formation of Sb^3+^-related low-energy STEs^39,40^. This assumption was supported by the appreciably higher distortion indices (*D*_*dis*_) of octahedron units in Cs_2_NaScCl_6_ than Cs_2_NaGdCl_6_ upon Sb^3+^ doping (Fig. S21).

We conducted density functional theory (DFT) calculations of the electronic structures to shed more light on the STE-derived optical property. The band structure of Cs_2_NaScCl_6_ featured a direct bandgap at the *Γ* point with an energy of 3.91 eV, where the conduction band minimum (CBM) and valence band maximum (VBM) mainly derived from Sc 3d and Cl 3p orbitals, respectively (Fig. 3f). The introduction of Sb^3+^ modifies the local electronic structures by forming extra inter-electronic states, where Cl 3p-hybridized Sb^3+^ 5s and 5p orbitals respectively appeared at 1.02 and 0.62 eV above the original VBM and CBM, contributing to the formation of Sb^3+^-related dual STE emissions (Fig. 3g and Fig. S22). Remarkably, as-prepared Cs_2_NaScCl_6_:Sb^3+^ crystal featured excellent thermal and air stability, offering great superiorities for constructing high-quality light-emitting devices (Figs. S23–24).

### X-ray-excited multiexcitonic emissions

**Fig. 4:**
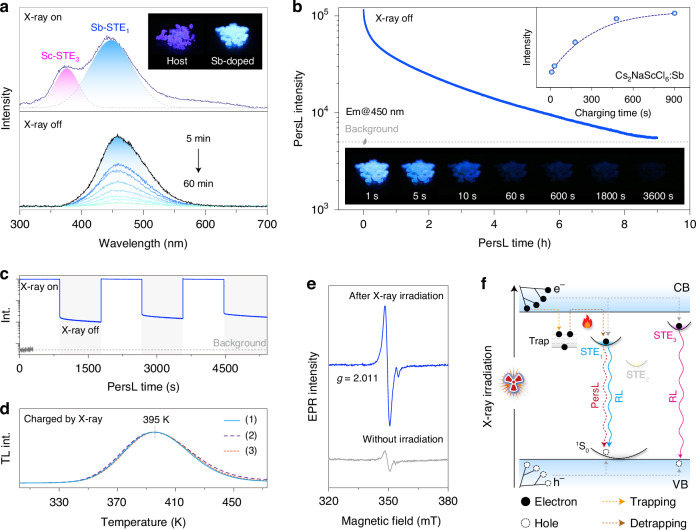
X-ray-excited multiexcitonic emissions **a** RL spectra of Cs_2_NaScCl_6_:Sb^3+^ (0.75%) crystal under X-ray irradiation (voltage: 50 kV, dose rate: 4.5 mGy·s^−1^), along with PersL spectra after the cessation of X-ray for 5–60 min. Insets show the RL photographs of bare and Sb^3+^-doped crystals. **b** PersL decay profile and photographs of Cs_2_NaScCl_6_:Sb^3+^ crystal after X-ray charging for 15 min. Note that the PersL decay profile was recorded after a short delay of 10 min. Inset shows the initial PersL intensity recorded immediately after the cessation of X-ray irradiation as a function of charging time. **c** Repeated PersL decay profile and **d** TL spectra of Cs_2_NaScCl_6_:Sb^3+^ crystal after X-ray charging. **e** EPR spectra of as-prepared Cs_2_NaScCl_6_:Sb^3+^ crystal recorded at RT. The sample was measured before and after X-ray irradiation. Note that *g*-factor accounts for the coupling between orbital and spin angular momentum. The *g* value was around 2.011 for the crystal, which possibly originated from native defects formed during synthesis^48^. **f** Proposed mechanism of possible X-ray-activated RL and PersL process. Note that the trap states are bound with Sb^3+^ due to the absence of PersL in the host crystal, and yellow emission is extremely weak under high-energy excitation due to the relatively low energy of the STE state

The extremely high PLQYs of multiexcitionic emissions endow Cs_2_NaScCl_6_:Sb^3+^ crystal with efficient radioluminescence (RL) performance. Under X-ray irradiation, the as-prepared crystal emitted intense violet-blue light with a relatively high light yield (~11,000 photons·MeV^−1^), low detection limit (~26.2 nGys^−1^), and high irradiation stability (Fig. S25). Notably, RL spectra of Cs_2_NaScCl_6_:Sb^3+^ crystals displayed an additional broadband emission in the range of 350–400 nm alongside the Sb^3+^-induced STE emission (Fig. 4a). Such high-energy emission might be attributed to the recombination of localized excitons bound with Sc^3+^-related polyhedron upon high-energy excitation (e.g., X-ray or synchrotron irradiation), which is hardly accessible by UV excitation^41,42^. This speculation was supported by the appearance of similar emission in Sc^3+^-contained halide perovskites (e.g., Cs_2_NaLuCl_6_:Sc^3+^, Cs_2_NaScCl_6_:Ag^+^/Bi^3+^, and Cs_2_ScCl_5_·H_2_O:Sb^3+^) upon X-ray excitation (Fig. S26).

After ceasing X-ray excitation, we observed persistent luminescence (PersL) in Sb^3+^-doped transparent crystal that showed a close spectral resemblance to blue emission under 335 nm excitation (Fig. 4a). The undetectable PersL phenomenon in bare crystal verified the main contribution of Sb^3+^-induced high-energy STE recombination to the PersL. By prolonging the charring time, the initial PersL intensity steadily increased and reached a plateau at a charging time of 15 min (Inset of Fig. 4b). Remarkably, we recorded an impressively long PersL lifetime exceeding 9 h after X-ray irradiation for 15 min (Fig. 4b). The highly transparent nature of the single crystal enabled the high-brightness PersL output by mitigating the scattering and self-absorption, outperforming the performance of the existing Sb^3+^-doped PersL materials (Table S3). Moreover, the PersL behavior showed high repeatability under repetitive charging measurements, demonstrating the high photostability of as-prepared single crystals against X-ray irradiation (Fig. 4c).

To gain mechanistic insight into the PersL process, the Cs_2_NaScCl_6_:Sb^3+^ crystal was assessed by thermoluminescence (TL) measurement. After X-ray charging, the TL spectrum showed a single broadband in the range of 340–470 K. Based on the initial-rise analysis, the trap depths were estimated to be around 0.51–0.56 eV (Fig. 4d and Fig. S27)^43^. This narrow trap distribution indicates the presence of a single PersL-active trap state in the Cs_2_NaScCl_6_:Sb^3+^ crystal. The trap-filling process was substantially promoted by prolonging the X-ray irradiation time, as revealed by the boosted TL intensity (Fig. S28). The nearly identical TL profiles under repeated measurements indicated that the long-term high-energy irradiation hardly created new trap states in as-prepared crystals. Moreover, the capture of charge carriers at trap states was verified by electronic paramagnetic resonance (EPR) spectroscopy (Fig. 4e). The intensity of the original EPR signal in the crystal was greatly enhanced upon X-ray charging, in consistency with the TL results. These observations indicated that the PersL process in Cs_2_NaScCl_6_:Sb^3+^ crystal was dominated by trap states, which can realize long-lived electron trapping under the irradiation of high-momentum X-ray photons. The appearance of a single blue peak in the PersL spectrum suggested that the trapped electrons were mainly released to the STE_1_ center, probably through the local de-trapping mechanism (Fig. 4f)^44^. Our control experiments revealed that electron filling in the trap states required high charging energy, which explained the absence of PersL after UV excitation (Fig. S29).

### Mechanically excited multiexcitonic emissions

**Fig. 5:**
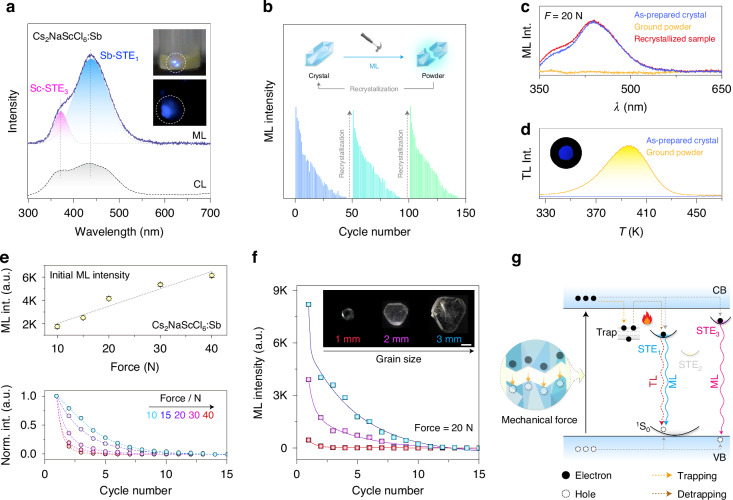
Mechanically excited multiexcitonic emissions **a** ML and CL spectra of Cs_2_NaScCl_6_:Sb^3+^ (0.75%) crystal. Insets show the ML photographs of the crystal in the ambient light or dark under continuous grinding with a glass rod. **b** The cyclic stability of ML intensity in Cs_2_NaScCl_6_:Sb^3+^ crystal in the dark under continuous grinding and recrystallization treatment. Inset shows the schematic of reproducible ML. **c** ML spectra of as-prepared crystal, ground powder, and recrystallized Cs_2_NaScCl_6_:Sb^3+^. **d** TL spectra of as-prepared crystal and ground powder sample, and inset shows the TL image of the ground sample at around 395 K. **e** Initial ML intensity versus applied force for Cs_2_NaScCl_6_:Sb^3+^ crystal (top panel) and corresponding intensity decay profile (bottom panel). **f** ML intensity of composite films versus the grain size of single crystals under continuous mechanical excitation at 20 N. Insets show the photographs of millimeter-sized single crystals under daylight conditions. Scale bar = 1 mm. **g** Proposed mechanism of possible luminescence process in Cs_2_NaScCl_6_:Sb^3+^ crystal under mechanical excitation

Strikingly, we observed bright luminescence from as-synthesized Cs_2_NaScCl_6_:Sb^3+^ crystal during grinding action, which was even visible to the naked eyes in ambient light (Video S1). Under mechanical excitation, the ML spectrum was mainly composed of two high-energy broadband STE emissions, in close resemblance to X-ray-excited RL spectra (Fig. 5a and Fig. S30). The repeatability test over continuous grinding cycles revealed a gradual drop in ML intensity as the single crystals were fragmented into powders (Fig. 5b, c and Video S2). As a result, the ground powder sample gradually lost its ML property, which could hardly recover by UV charging (Fig. S31). Nevertheless, reproducible ML can be realized through a simple recrystallization process (Fig. S32). Notably, the mechanical action also resulted in the population of the trap states according to TL analysis, although the trap-filling efficiency was insufficient to induce detectable PersL (Fig. 5d).

To quantify the ML property, we encapsulated a uniform mixture of crystals and polydimethylsiloxane (PDMS) elastic matrix in a transparent thermoplastic polyester (polyethylene glycol terephthalate, PET) with good mechanical strength. We found a linear dependence of initial ML intensity on the applied force, and a sharper decline in ML intensity was detected under successive mechanical action at a higher load (Fig. 5e). Moreover, the ML was substantially enhanced by increasing the grain size of single crystal (Fig. 5f). Similar ML behavior was also observed in Sb^3+^-doped Cs_2_NaRECl_6_ (RE = Lu, Y, and Gd) crystals with weaker ML intensities, likely due to their lower PLQYs compared to Sc-based crystals (Fig. S33).

The strong correlation between the ML behavior and the fracture of single crystals indicates a fractoluminescence mechanism (Fig. 5g). During the fracture process, charge separation occurs on the newly created surfaces due to the breaking of chemical bonds. The following charge recombination induces direct excitation of the luminescent center by electron bombardment^45,46^. Our assumption was validated by the similar spectral profiles between ML and cathodoluminescence (CL) of as-prepared single crystals (Fig. 5a and Fig. S30). The ability of fast-moving electrons to excite various luminescent centers in crystals, coupled with the high doping capacity of the Sc^3+^ site, enabled unprecedented ML tuning from UV-C to NIR within Cs_2_NaScCl_6_ crystals using different dopant ions (Figs. S34–35 and Video S3).

### Excitation-mode-selective emission for information security

**Fig. 6:**
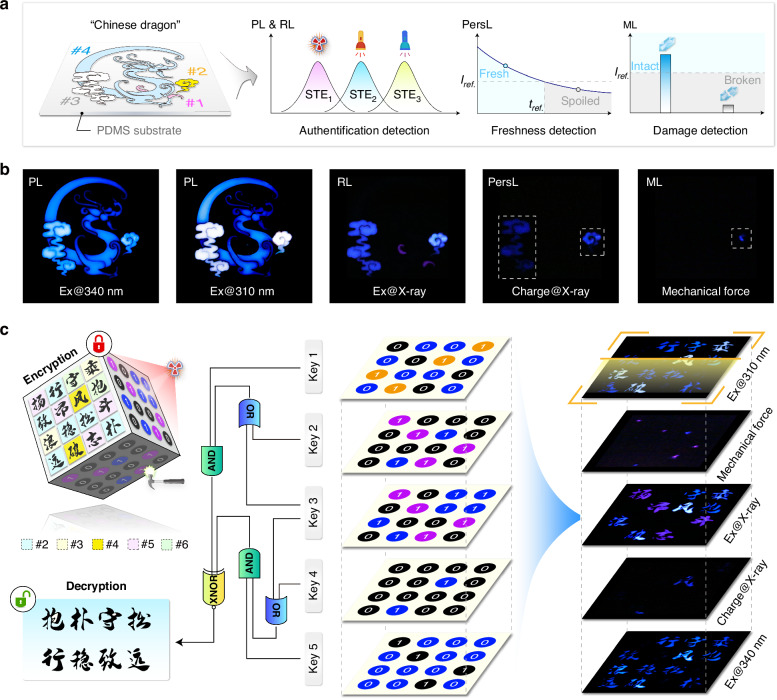
Multi-level authentication and information encryption **a** Design principle of the “Chinese dragon” security pattern composed of crystals with different compositions and grain sizes. The pattern permits product authentification and transportation history tracking based on the excitation-selective STE emissions, X-ray charged PersL and ML behaviors. **b** Photographs of the pattern under different excitation modes and acquisition conditions. Note that the PersL image was taken after X-ray charging for 15 min. **c** Schematic of logic encryption through individually addressable emission features of the as-prepared crystals. Note that the white emission unit under 310 nm excitation, the bright unit under mechanical force (ML) and X-ray irridation (RL and PersL), and the dark unit under 340 nm excitation are designated as binary data “1” for logic operations. Samples used for generating these patterns are #1: Cs_2_NaScCl_6_ fine powder; #2 Cs_2_NaInCl_6_:Sb^3+^ (0.75%) fine powder; #3: Cs_2_NaScCl_6_:Sb^3+^ (0.75%) fine powder; #4: Cs_2_NaScCl_6_:Sb^3+^ (0.75%) single crystal (grain size~2 mm); #5: Cs_2_NaScCl_6_ single crystal (grain size~2 mm); #6: Cs_2_NaLuCl_6_:Sb^3+^ (0.75%) fine powder

The ability to manipulate switchable multiexcitonic emissions using different excitation modes offers unique opportunities for multi-level authentication technology (Fig. S36). In an illustrative design, we prepared a security label for product authentication using four types of double perovskite crystals with distinct optical characteristics (Fig. 6a). By controlling the excitation wavelength, variable graphic information could be revealed as security codes due to color switching in selective blocks of the pattern. More importantly, the time or impact history experienced by the products during transportation can be quantitatively assessed by the residual PersL or ML intensity of the label at prescribed positions (Fig. 6b). These results substantiated the great superiorities of the excitation-selective multiexcitonic luminescence for intelligent authentication technology, with inherent features of high confidentiality, direct visualization, and all-in-one functionality.

In a further set of experiments, we established a pattern technique for logic encryption by leveraging the highly variable and individually addressable emission features of the crystals. In our design, the pattern was examined multiple times under specific excitation/acquisition conditions to generate a series of optical outputs, which were then subjected to predetermined logic operations to reveal the cryptographic data. As depicted in Fig. 6c, we demonstrated that programmable optical codes could be obtained using five sets of crystals. Accordingly, eight Chinese characters were extracted as the decrypted information from a background of sixteen through successive OR, AND, and XNOR operations. Notably, patterns made of small-sized crystals may lose their ML property after being subjected to mechanical action, offering one-off authentication as an extra level of encryption.

## Discussion

In conclusion, we have developed a class of transparent all-inorganic halide double perovskite crystals featuring efficient multiexcitonic emissions that can be selectively activated by excitations of ultraviolet light, X-ray irradiation, and mechanical action. The excitation-mode-selective luminescence stemmed from the distinct properties of STEs associated with spatially separated [ScCl_6_]^3−^ and [SbCl_6_]^3−^ units. Remarkably, we recorded efficient white PL emission (PLQY >97%), long-lasting blue PersL (duration >9 h), and self-reproducible blue-violet ML in a single component of crystal. These advances may inspire new design principles of advanced metal halide perovskite crystals with a broadened range of applications, such as information protection with high-level security.

## Materials and methods

### Preparation of lead-free metal halide perovskite crystals

All samples, including Cs_2_NaRECl_6_ doped with different ions, Cs_2_ScCl_5_·H_2_O:Sb^3+^, and Cs_3_Sb_2_Cl_9_:Sb^3+^, were grown via a modified hydrothermal process. Additional experimental details are provided in the Supplementary Information.

### Preparation of composite ML films

To quantify the ML properties of samples, two transparent polyethylene glycol terephthalate (PET) sheets (1.5 cm × 1 cm) were employed to encapsulate a uniform mixture of single crystals (0.3 g) and polydimethylsiloxane (PDMS, 0.3 g).

### Fabrication of white light-emitting diode (WLED) devices

The WLED devices were fabricated by combining the white-emitting crystals with UV-LED chips (310 nm). Firstly, the phosphors were thoroughly mixed with silicone paste and then coated on the surface of commercial LED chips. Finally, the WLED devices were manufactured after being cured at 80 °C for 1 h.

### Characterization

The powder X-ray diffraction (PXRD) patterns were acquired by a Bruker D8 Advance powder diffractometer at 40 kV and 40 mA with Cu-Kα (*λ* = 1.54056 Å) irradiation, and the crystal structure refinements were analyzed using the General Structure Analysis System (GASA-II). The microstructural and elemental characterizations, including scanning electron microscopy (SEM), energy dispersive spectrometer (EDS), high-resolution transmission electron microscopy (HR-TEM), and selected area electron diffraction (SAED), were conducted on a Novanano-450 field SEM and a JEM-ARM200F TEM. X-ray photoelectron spectroscopy (XPS) was recorded by a Thermo Fisher ESCALAB 250Xi. Raman spectroscopy was performed using an HR Evolution confocal Raman microscopy. Thermogravimetric analysis (TGA) curve was recorded using a Hitachi STA7300 at a heating rate of 10 °C·min^−1^. The inductively coupled plasma mass (ICP-MS) spectrum was measured using an ion mass spectrometer (Agilent 7800). The optical properties of the samples at different temperatures, including photoluminescence emission (PL)/excitation (PLE) spectra, time-resolved decay curves, and photoluminescence quantum efficiency (PLQY), were measured using a Horiba FL3 fluorescence spectrometer equipped with a 450 W xenon lamp as the excitation sources. Ultraviolet-visible (UV-vis) absorption spectra were collected by a Hitachi 4100 UV-Vis-NIR spectroscopy. The radioluminescence (RL) property was measured using a fiber optic spectrometer of Ocean Optics QE65pro (200–1100 nm) coupled with an integrating sphere and an X-ray tube (50 kV, 5–70 μA Amptek Inc, Mini-X). The persistent luminescence (PersL) decay curves were recorded using an FS5 spectrometer (Edinburgh Instruments) coupled with an X-ray tube (50 kV, Tungsten target, Moxtex). Note that the X-ray outlet was set to 1 cm away from the sample for all spectral measurements. Thermoluminescence (TL) spectra were recorded using a sensitive power meter (1936-R, Newport) and FJ-427A1 TL dosimeter at a heating rate of 2.5 K·s^−1^. Electron paramagnetic resonance (EPR) spectra of the samples were measured using a Bruker EMX-PLUS EPR spectrometer, at a microwave frequency of 9.8 GHz, a microwave power of 2 mW, a magnetic field modulation amplitude of 4G, a magnetic field modulation frequency of 100 kHz, and a time constant of 30 s. The ML performance of samples was assessed quantitatively by a home-built measuring apparatus according to our previous work, including a linear motor, a digital push-pull gauge, and a fiber optic spectrometer^10^. Note that all the error bars of ML intensity represent the standard deviations from three sets of repeated measurements. Cathodoluminescence (CL) spectra were recorded by a GATAN MonoCL4 cathode fluorescence spectrometer attached to a field-emission SEM instrument. Photographs of single crystals were taken with an Olympus BX53 optical microscope. PersL and ML images were recorded using a digital camera (Canon 90D) and an NIR camera.

### Computational methodology

The theoretical calculations were performed by the Cambridge Sequential Total Energy Package (CASTEP) and Vienna *Ab* Initio Simulation Package (VASP). The formation energy was calculated by density functional theory (DFT) in the form of generalized gradient approximation (GGA) Perdew-Burke-Ernzerhof (PBE) function, and DFT was employed for the band structure and partial density of states (PDOS)^47^. A Monkhorst-Pack 5 × 5 × 5 k mesh was used as the Brillouin zone, and the kinetic energy cutoff and self-consistent field (SCF) were set as 550 eV and 10^−5^ eV·atom^−1^, respectively.

## Supplementary information

Supplementary Information

Supplementary Video S1

Supplementary Video S2

Supplementary Video S3

## Data Availability

All relevant data that support the findings of this work are available from the corresponding author on reasonable request.
