# Supplementary material for: Excitation-mode-selective emission through multiexcitonic states in a double perovskite single crystal
Source: Light Sci Appl. 2025 Jan 2;14:21. doi: 10.1038/s41377-024-01689-7 (PMC11693751; doi:10.1038/s41377-024-01689-7)
Supplement: Supplementary file 1 — Supplementary Information [file 41377_2024_1689_MOESM1_ESM.docx]

**Supplementary Information for**

**Excitation-Mode-Selective Emission through Multiexcitonic States in a Double Perovskite Single Crystal**

Hao Suo^1,2#^*, Nan Wang^1#^, Yu Zhang^1#^, Xin Zhang^2^, Jinmeng Xiang^3^, Xiaojia Wang^4^, Guansheng Xing^5^, Dongxu Guo^1^, Jiwen Chang^1^, Yu Wang^1^, Panlai Li^1^, Zhijun Wang^1^, Yuhai Zhang^4^, Bing Chen^5^, Shuzhou Li^6^, Chongfeng Guo^3^* & Feng Wang^2^*

Correspondence: Hao Suo (suo@hbu.edu.cn) or Chongfeng Guo (guocf@nwu.edu.cn), or Feng Wang (fwang24@cityu.edu.hk)

^1^ Hebei Key Laboratory of Optic-electronic Information and Materials, College of Physics Science & Technology, Hebei University, Baoding 071002, China.

^2^ Department of Materials Science and Engineering, City University of Hong Kong, Kowloon 999077, Hong Kong SAR, China.

^3^ State Key Laboratory of Photon-Technology in Western China Energy, Institute of Photonics & Photon-Technology, Northwest University, Xi'an 710127, China.

^4^ Institute for Advanced Interdisciplinary Research, University of Jinan, Jinan, Shandong 250022, China.

^5^ College of Electronic and Optical Engineering & College of Flexible Electronics (Future Technology), Nanjing University of Posts and Telecommunications, Nanjing 210023, China.

^6^ School of Materials Science and Engineering, Nanyang Technological University, 50 Nanyang Avenue, Singapore, 639798 Singapore.

^#^ These authors contributed equally: Hao Suo, Nan Wang, Yu Zhang

**I. Supplementary Methods**

**1. Raw materials and reagents.** Cesium chloride (CsCl, 99.99%), antimony chloride (SbCl_3_, 99.99%), scandium chloride hexahydrate (ScCl_3_·6H_2_O, 99.99%), yttrium chloride hexahydrate (YCl_3_·6H_2_O, 99.99%), gadolinium chloride hexahydrate (GdCl_3_·6H_2_O, 99.90%), indium chloride (InCl_3_, 99.99%), gallium chloride (GaCl_3_, 99.99%), cerium chloride (CeCl_3_, ≥99.99%), terbium chloride hexahydrate (TbCl_3_·6H_2_O, 99.90%), lanthanum chloride hexahydrate (LaCl_3_·6H_2_O, 99.99%), thulium chloride hexahydrate (TmCl_3_·6H_2_O, 99.99%), chromium chloride hexahydrate (CrCl_3_·6H_2_O, 98%), ytterbium chloride hexahydrate (YbCl_3_·6H_2_O, 99.99%), bismuth chloride (BiCl_3_, 99.99%), silver chloride (AgCl, 99.5%), sodium chloride (NaCl, 99.99%), manganese chloride (MnCl_2_, ≥ 99%), scandium acetate hydrate (ScC_6_H_9_O_6_·xH_2_O, 99.99%-Sc), scandium oxide (Sc_2_O_3_, 99.99%), alumina (Al_2_O_3_, 99.99%), samarium oxide (Sm_2_O_3_, 99.99%), europium oxide (Eu_2_O_3_, 99.99%), praseodymium oxide (Pr_6_O_11_, 99.99%), dysprosium oxide (Dy_2_O_3_, 99.90%), holmium oxide (Ho_2_O_3_, 99.99%), erbium oxide (Er_2_O_3_, 99.99%), neodymium oxide (Nd_2_O_3_, 99.99%), lutetium oxide (Lu_2_O_3_, 99.99%), and isopropyl alcohol (C_3_H_8_O, 99.5%) were purchased from Aladdin Biochemical Technology Co., Ltd. Scandium oxide (Sc_2_O_3_, 99.99%) was also purchased from Alfa Aesar for control experiments. Hydrochloric acid (HCl) was purchased from Tianjin Damao Chemical Reagent Factory. All chemicals and reagents were used as received without further purification.

**2. Preparation of doped Cs_2_NaScCl_6_ single crystals.** Millimeter-sized Cs_2_NaScCl_6_ single crystals doped with different ions were grown from concentrated hydrochloric acid via a modified hydrothermal process followed by cooling-induced crystallization. In a typical synthetic procedure of Cs_2_NaScCl_6_:Sb^3+^ crystal, 2 mmol CsCl, 0.8 mmol NaCl, and 0.5 mmol Sc_2_O_3_ were added to 0.85 mL of hydrochloric acid in a 25 mL Teflon-lined container. Note that the Cs/Sc/Na molar ratio was fixed as 2:0.8:1 to prevent the undesired formation of NaCl impurity. Subsequently, an additional HCl solution containing SbCl_3_ (0.75 mL; 0.01 M) was introduced into the above solution under vigorous stirring, followed by heating to 180 ℃ within 0.5 h and maintaining for 12 h. After cooling to room temperature (RT), the resulting crystals were collected and washed with isopropyl alcohol several times. Finally, the colorless transparent Cs_2_NaScCl_6_:Sb^3+^ single crystals were dried at 60 ℃ for 6 h and stored in a glass tube for subsequent characterizations. Note that the grain size of a single crystal can be tuned from 1 to 3 mm by controlling the cooling rate from 7 to 3 ℃·h^-1^**.** The synthesis procedures of Cs_2_NaRECl_6_ crystals (RE = Lu, Y, Gd) and Cs_2_NaScCl_6_ crystals doped with other ions (Bi^3+^, Ga^3+^, Al^3+^, In^3+^, Mn^2+^, Cr^3+^, Lu^3+^, Yb^3+^, Tm^3+^, Er^3+^, Ho^3+^, Y^3+^, Dy^3+^, Tb^3+^, Gd^3+^, Eu^3+^, Sm^3+^, Nd^3+^, Pr^3+^, Ce^3+^, and La^3+^) were similar except the introduction of corresponding raw materials in the first step.

**3. Recrystallization of Cs_2_NaScCl_6_:Sb^3+^ single crystals.** After grinding the single crystals into the fine powder, the resulting samples can be recrystallized into single crystals via a repeated hydrothermal synthesis. In a typical procedure, 1 mmol of fine powder was weighed and added to 1.6 mL of hydrochloric acid in a 25 mL Teflon-lined container under vigorous stirring. After being maintained at 180 ℃ for 12 h, the resulting crystals were collected and washed with isopropyl alcohol several times. Finally, the colorless transparent single crystals were obtained after drying at 60 ℃ for 6 h.

**4. Preparation of Sb^3+^ doped Cs_2_ScCl_5_·H_2_O single crystals.** According to previous work, Cs_2_ScCl_5_·H_2_O:Sb^3+^ crystals were synthesized via a modified hydrothermal process^1^. In a typical synthetic procedure, 2 mmol CsCl, 0.4962 mmol Sc_2_O_3_ and 0.0075 mmol SbCl_3_ were added to 2 mL of hydrochloric acid in a 25 mL Teflon-lined container under vigorous stirring. By incubation at 180 ℃ for 12 h, the resulting crystals were collected and washed with isopropyl alcohol several times. Finally, the transparent Cs_2_ScCl_5_·H_2_O:Sb^3+^ (0.75%) single crystals were dried at 60 ℃ for 6 h and stored in a glass tube for subsequent characterizations.

**5. Preparation of Sb^3+^ doped Cs_3_Sb_2_Cl_9_ single crystals.** In a typical synthetic procedure, 3 mmol CsCl and 2 mmol SbCl_3_ were added to 5 mL of hydrochloric acid in a 25 mL Teflon-lined container under vigorous stirring. After heating at 180 ℃ for 12 h, the resulting crystals were collected and washed with isopropyl alcohol several times. Finally, the transparent Cs_3_Sb_2_Cl_9_ single crystals were dried at 60 ℃ for 6 h and stored in a glass tube for subsequent characterizations.

**II. Supplementary Figures**

**
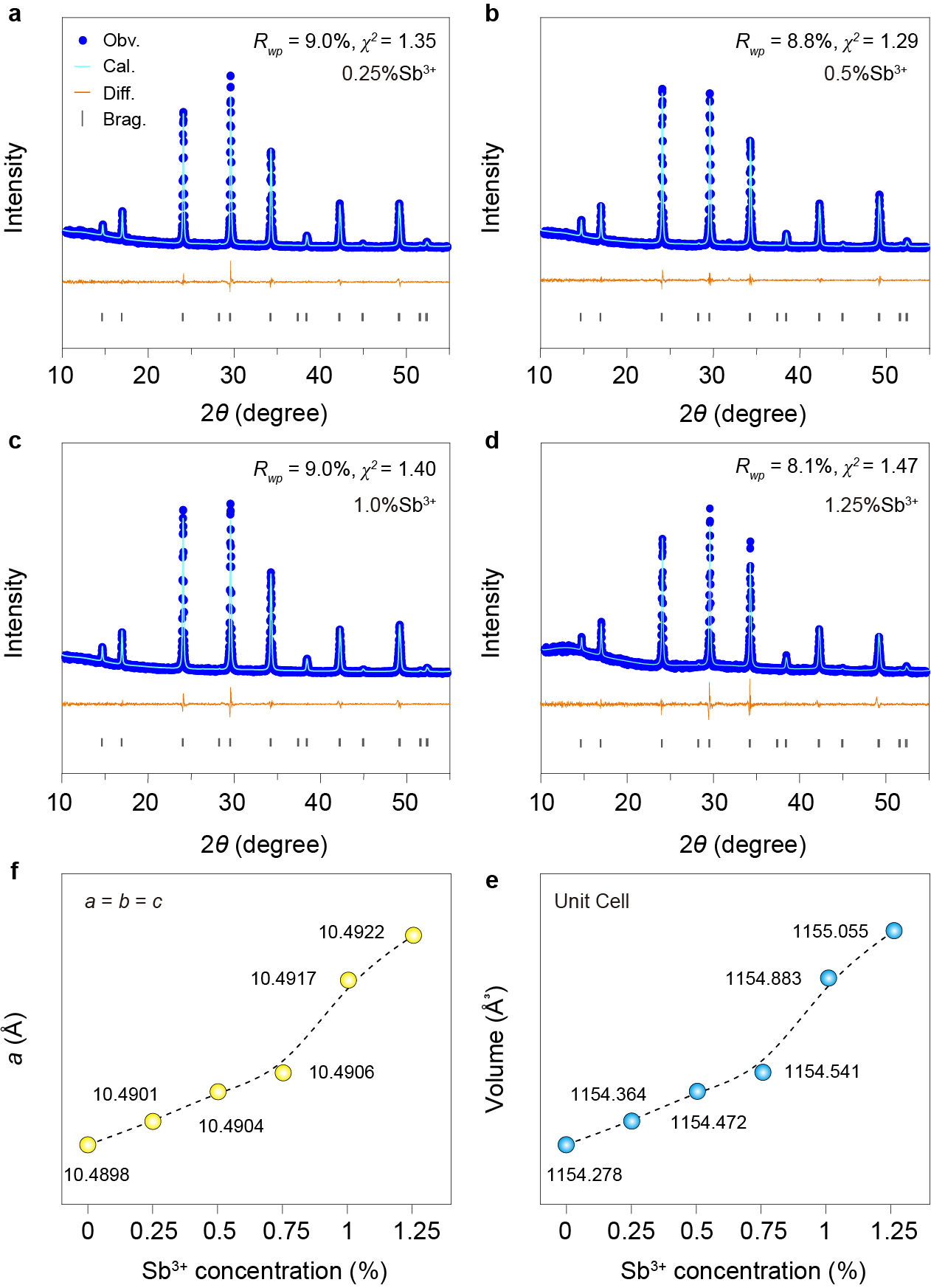
**

**Fig. S1 a-d**, Rietveld refinements of representative XRD patterns for Cs_2_NaScCl_6_:*x*Sb^3+^ (*x* = 0.25%, 0.5%, 1%, and 1.25%). **e-f**, Refined crystal parameter and unit cell volume. The calculated crystallographic structural parameters were summarized in **Table S2**. It can be found that the volume of the unit cell gradually increased with the increase of Sb^3+^ doping content, confirming the successful substitution of Sb^3+^ (0.76 Å) for smaller Sc^3+^ (0.745 Å).


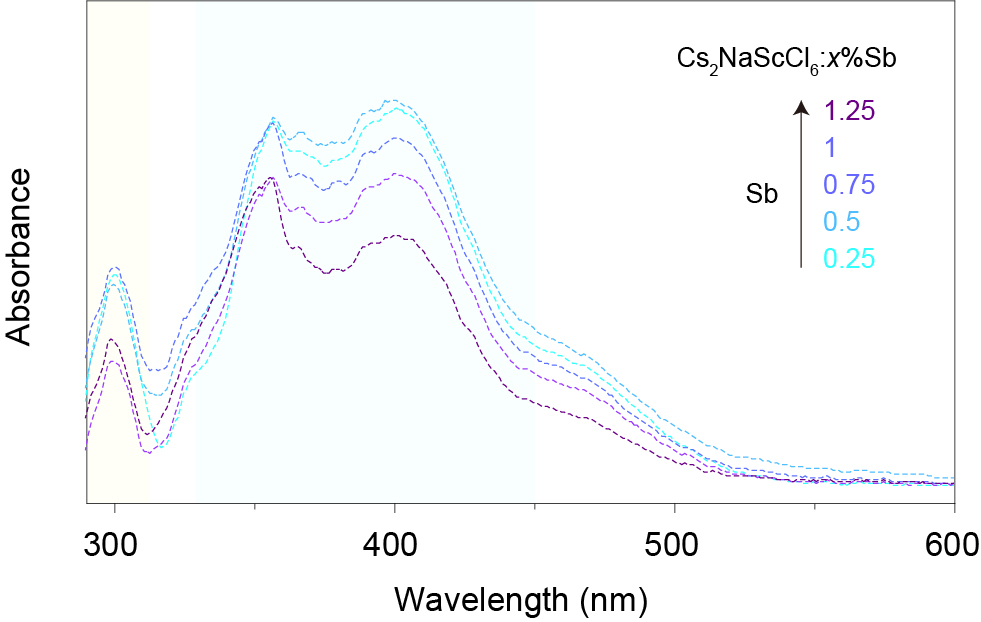


**Fig. S2** Absorption spectra of Cs_2_NaScCl_6_:*x*Sb^3+^ (*x* = 0.25–1.25%) crystals.


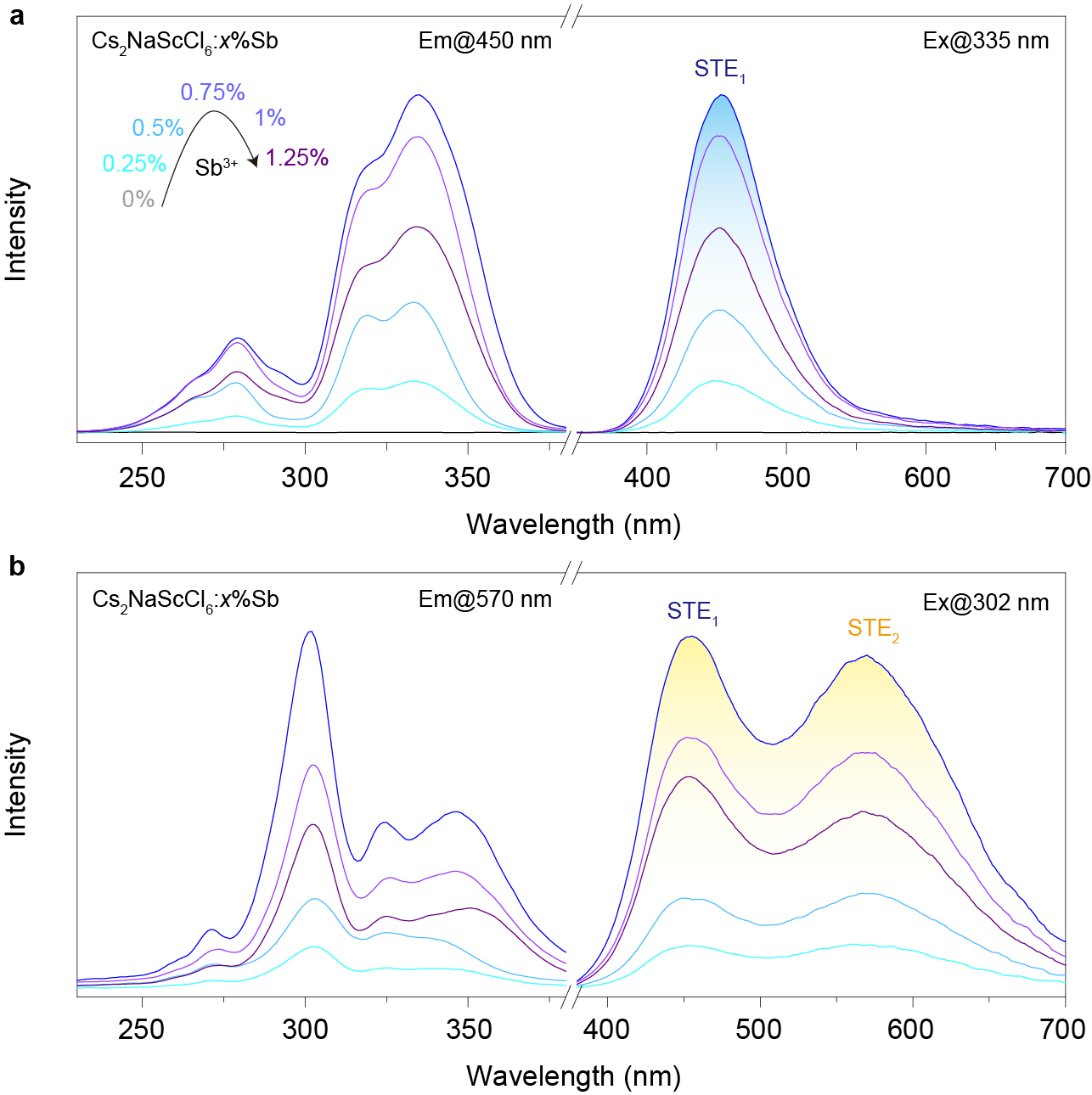


**Fig. S3 a**, PLE (*λ*_em_ = 450 nm) and PL (*λ*_ex_ = 335 nm) spectra of Cs_2_NaScCl_6_ crystals doped with different concentrations of Sb^3+^ ions (0–1.25%). **b**, PLE (*λ*_em_ = 570 nm) and PL (*λ*_ex_ = 302 nm) spectra of Cs_2_NaScCl_6_ crystals doped with different concentration of Sb^3+^ ions (0.25–1.25%). As Sb^3+^ concentration increased, both the blue and yellow emission bands gradually increased and then dropped after reaching the optimal value of 0.75%, while the band position and width hardly changed. Note that the bare Cs_2_NaScCl_6_ crystal emitted blue broadband light peaked at 450 nm under 335 nm excitation that was nearly identical to that of Sb^3+^-doped counterpart, while showed no emissions under 302 nm excitation.


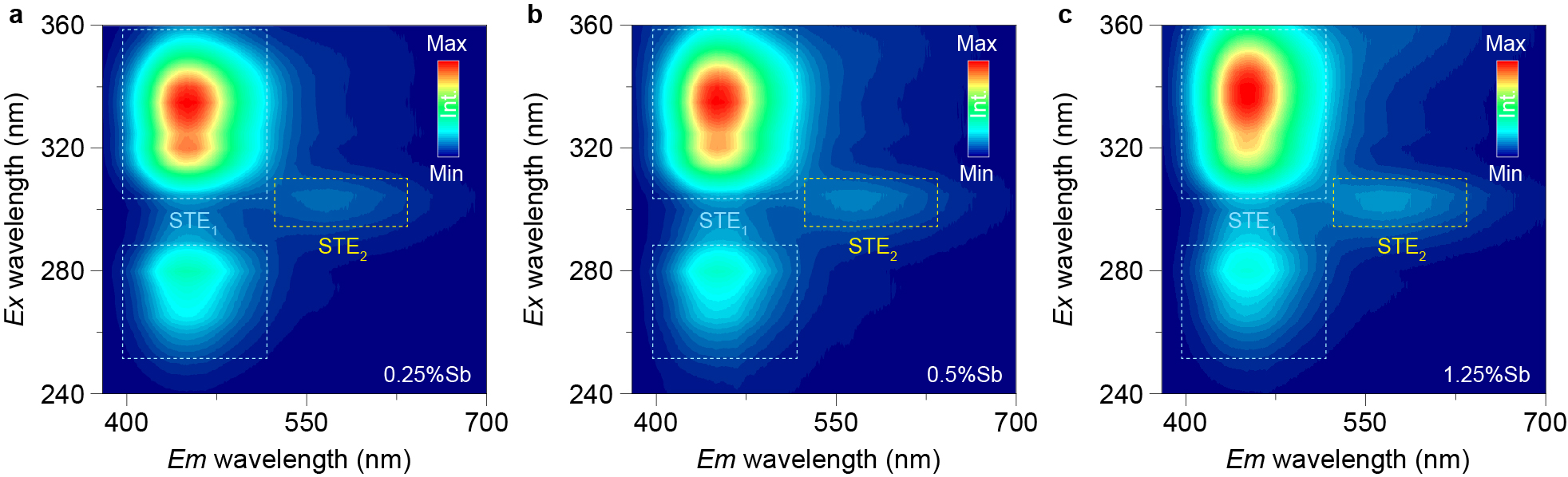


**Fig. S4** Contour plot of the excitation-wavelength-resolved PL spectra for Cs_2_NaScCl_6_ doped with different concentrations of Sb^3+^ ions (0.25%, 0.5%, and 1.25%) at RT. All these samples displayed similar PL profiles at different excitation wavelengths, indicating that the excitation-wavelength-controlled PL switching behavior is essentially independent of Sb^3+^ doping concentration.

**
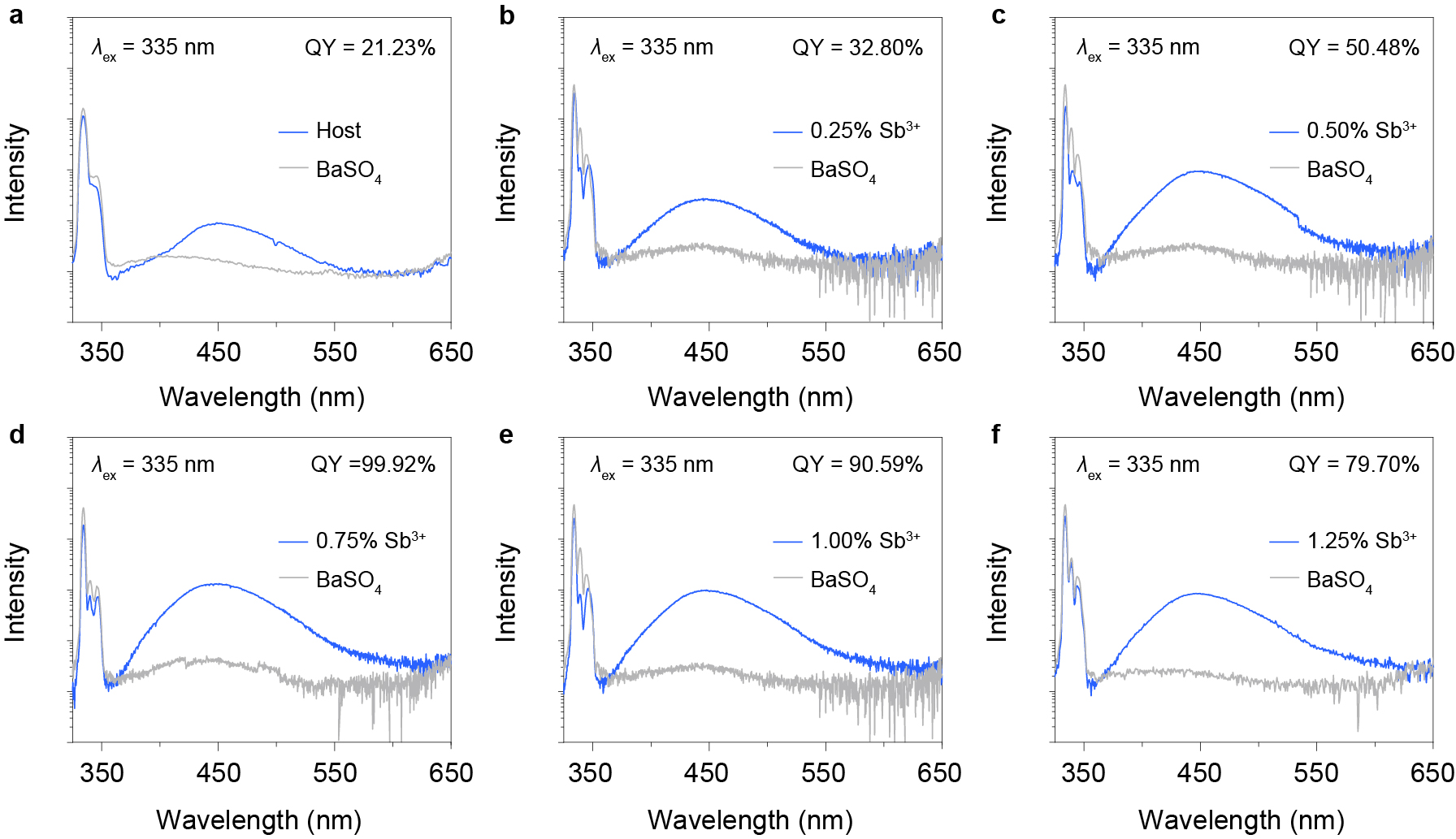
**

**Fig. S5** The PLQY values of Cs_2_NaScCl_6_ crystals doped with **a,** 0%, **b,** 0.25%, **c,** 0.50%, **d,** 0.75%, **e,** 1.00%, and **f,** 1.25% of Sb^3+^ under 335 nm excitation.

**
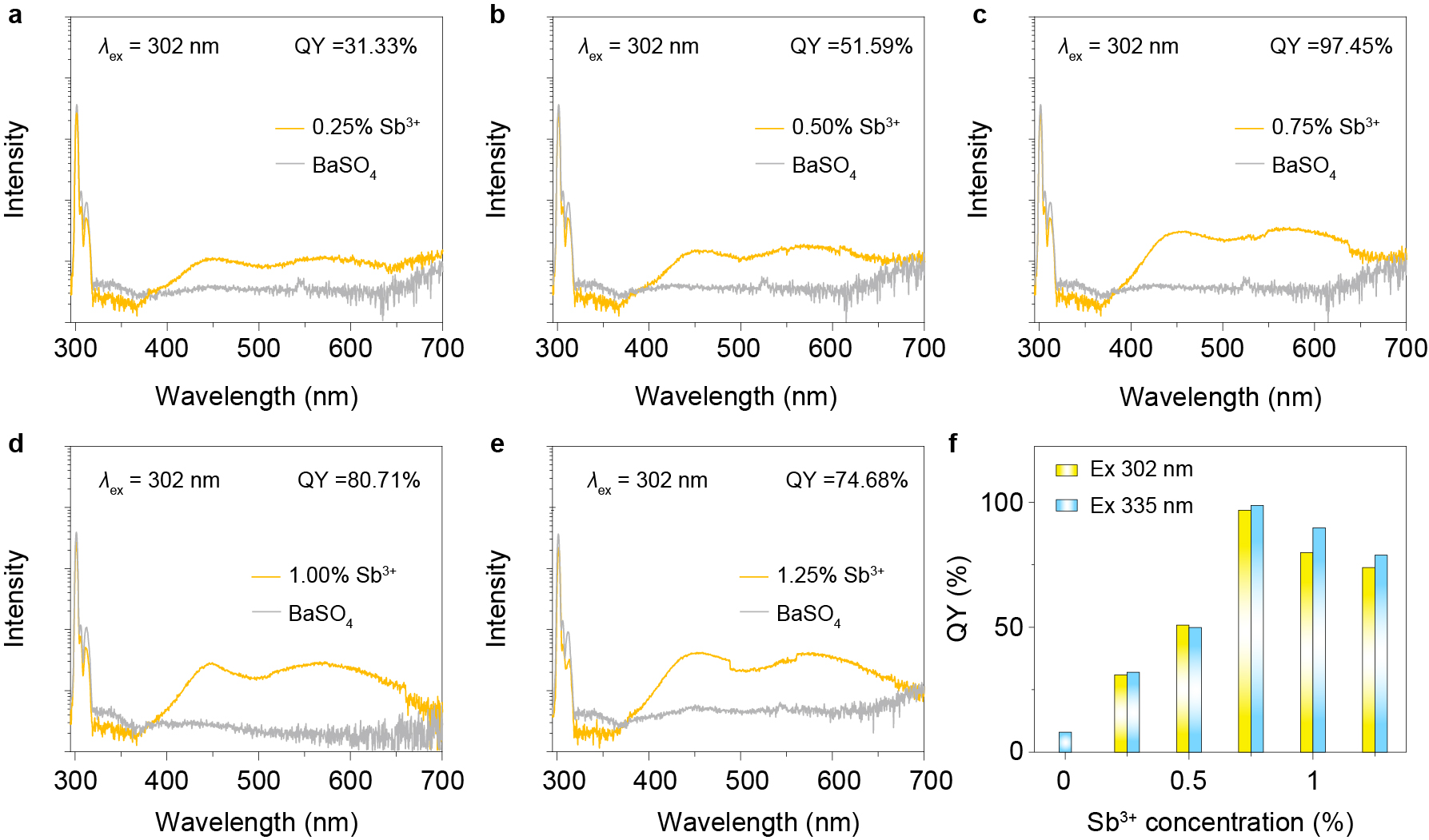
**

**Fig. S6** The PLQY values of Cs_2_NaScCl_6_ crystals doped with **a,** 0.25%, **b,** 0.50%, **c,** 0.75%, **d,** 1.00%, and **e,** 1.25% of Sb^3+^ under 302 nm excitation. **f,** PLQY values of blue and white broadband emissions as a function of Sb^3+^ doping concentration. Notably, the optimal PLQY values were determined to be around 99.9% and 97.5% for blue and white emissions in Cs_2_NaScCl_6_:Sb^3+^ (0.75%) crystal, respectively.

**
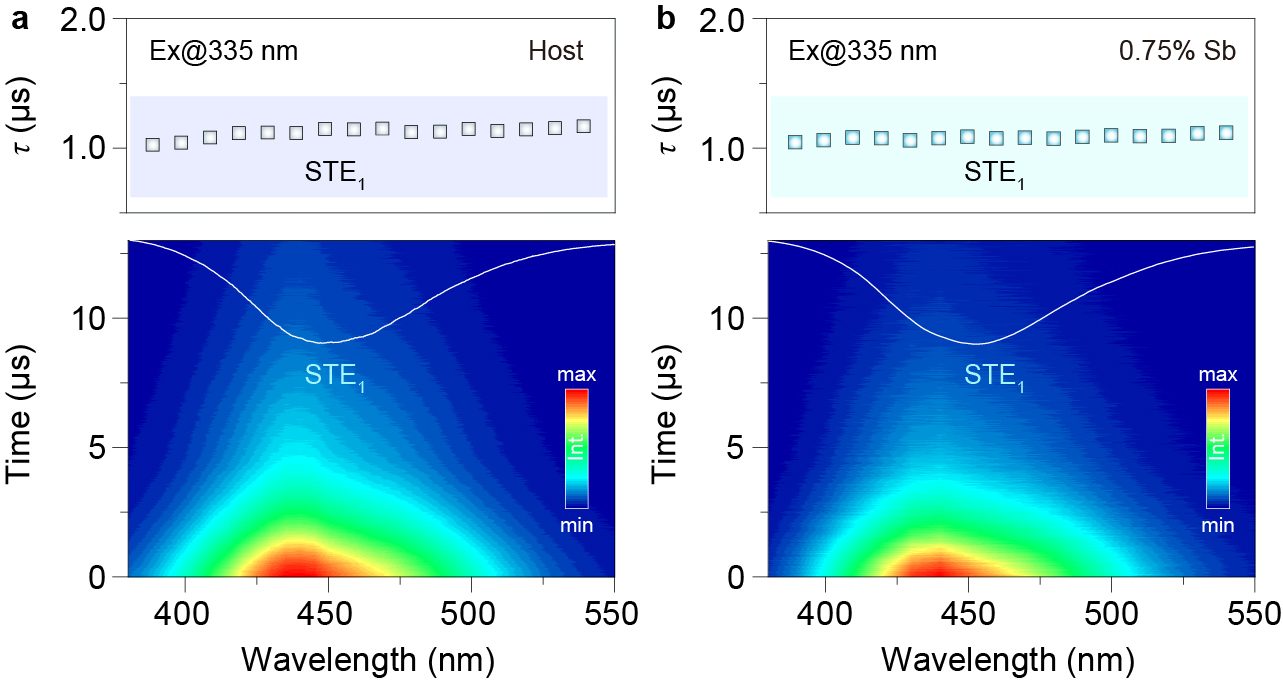
**

**Fig. S7** Time-resolved PL mapping and steady-state PL spectra under 335 nm excitation, along with the calculated lifetimes at different emission wavelengths of **a,** Cs_2_NaScCl_6_ and **b,** Cs_2_NaScCl_6_:Sb^3+^ (0.75%) crystals. Note that the transient spectroscopic behavior of blue STE emission in the bare crystal was nearly identical to the Sb^3+^-doped counterpart. The results indicated that a consistent luminescent center was populated under 335 nm excitation in these two samples.


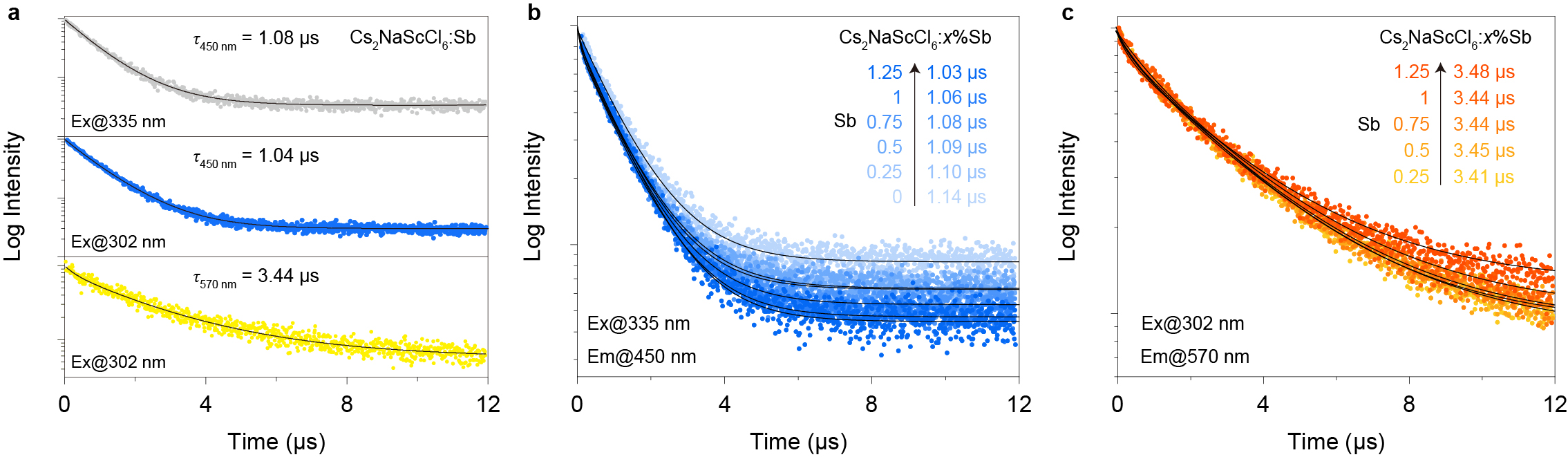


**Fig. S8 a,** Decay curves of blue emission at 450 nm in Cs_2_NaScCl_6_:Sb^3+^ (0.75%) crystal by 335 nm or 302 nm excitation (top and middle panels), along with yellow emission at 570 nm by 302 nm excitation (bottom panel). **b-c,** Decay curves of blue and yellow emissions as a function of Sb^3+^ concentrations. Note that all the decay curves can be well-fitted by a bi-exponential function with microsecond-scale lifetimes. The lifetime of the 450 nm emission hardly varied as the excitation wavelength was switched from 335 to 302 nm, indicating the negligible energy exchange between the two PL components due to the spatially separated polyhedral units. Moreover, the calculated lifetimes were essentially unaffected by the dopant concentration of Sb^3+^ ions.


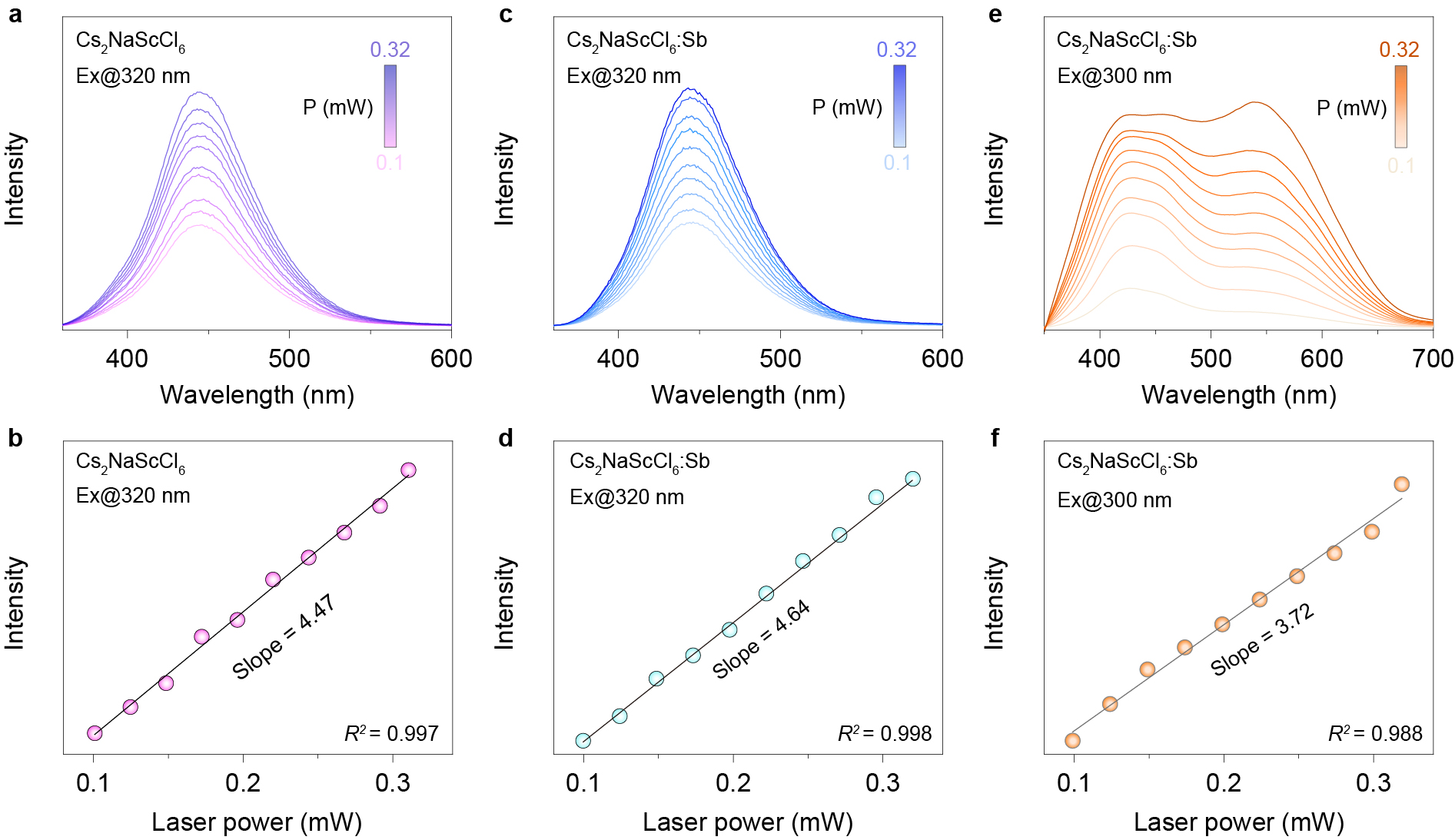


**Fig. S9** Excitation power density-dependent PL spectra and the corresponding integral intensity of **a-b,** Cs_2_NaScCl_6_ (*λ*_ex_ = 320 nm), **c-d,** Cs_2_NaScCl_6_:Sb^3+^ (0.75%, *λ*_ex_ = 320 nm), and **e-f,** Cs_2_NaScCl_6_:Sb^3+^ (0.75%, *λ*_ex_ = 302 nm) crystals in the range of 0.1–0.32 mW. It can be clearly observed that the PL intensity monotonously increased with increasing the laser power without saturation, thereby excluding the contribution of lattice defect states to the blue and yellow broadband emissions. Note that the excitation power density-dependent PL behavior of blue emission in the bare crystal was nearly identical to Sb^3+^-doped counterparts.


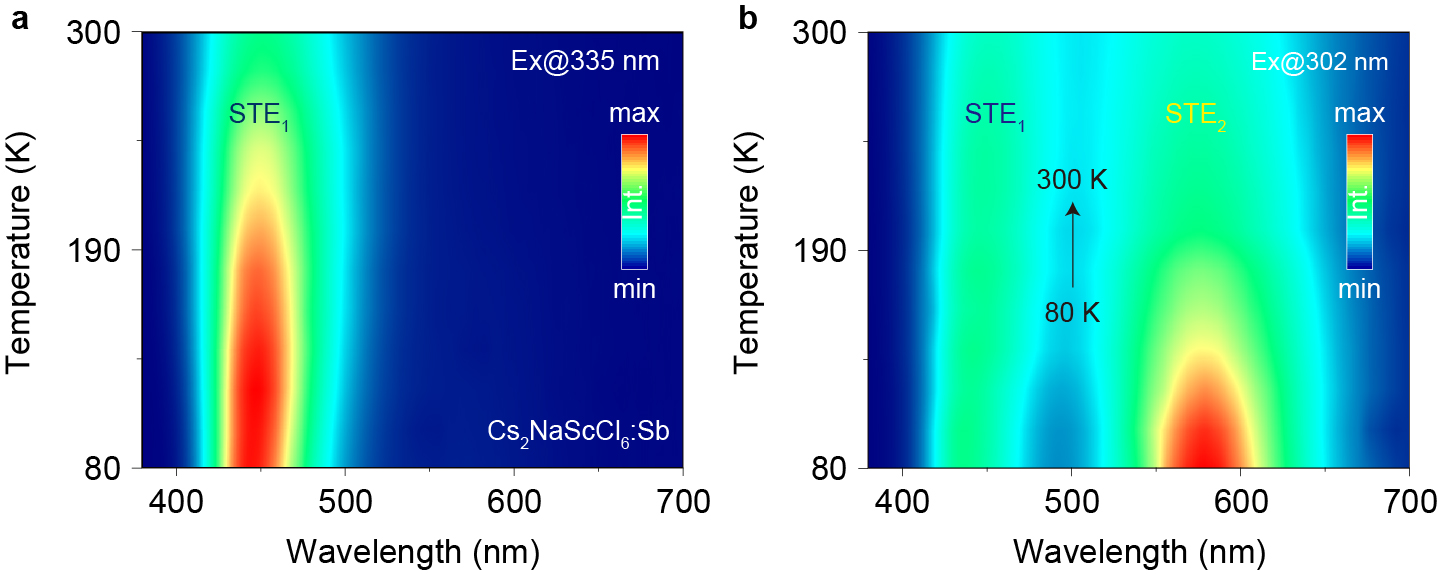


**Fig. S10** Contour plot of the temperature-dependent PL spectra (80–300 K) of Cs_2_NaScCl_6_:Sb^3+^ (0.75%) crystal under **a,** 335 and **b,** 302 nm excitation.

**
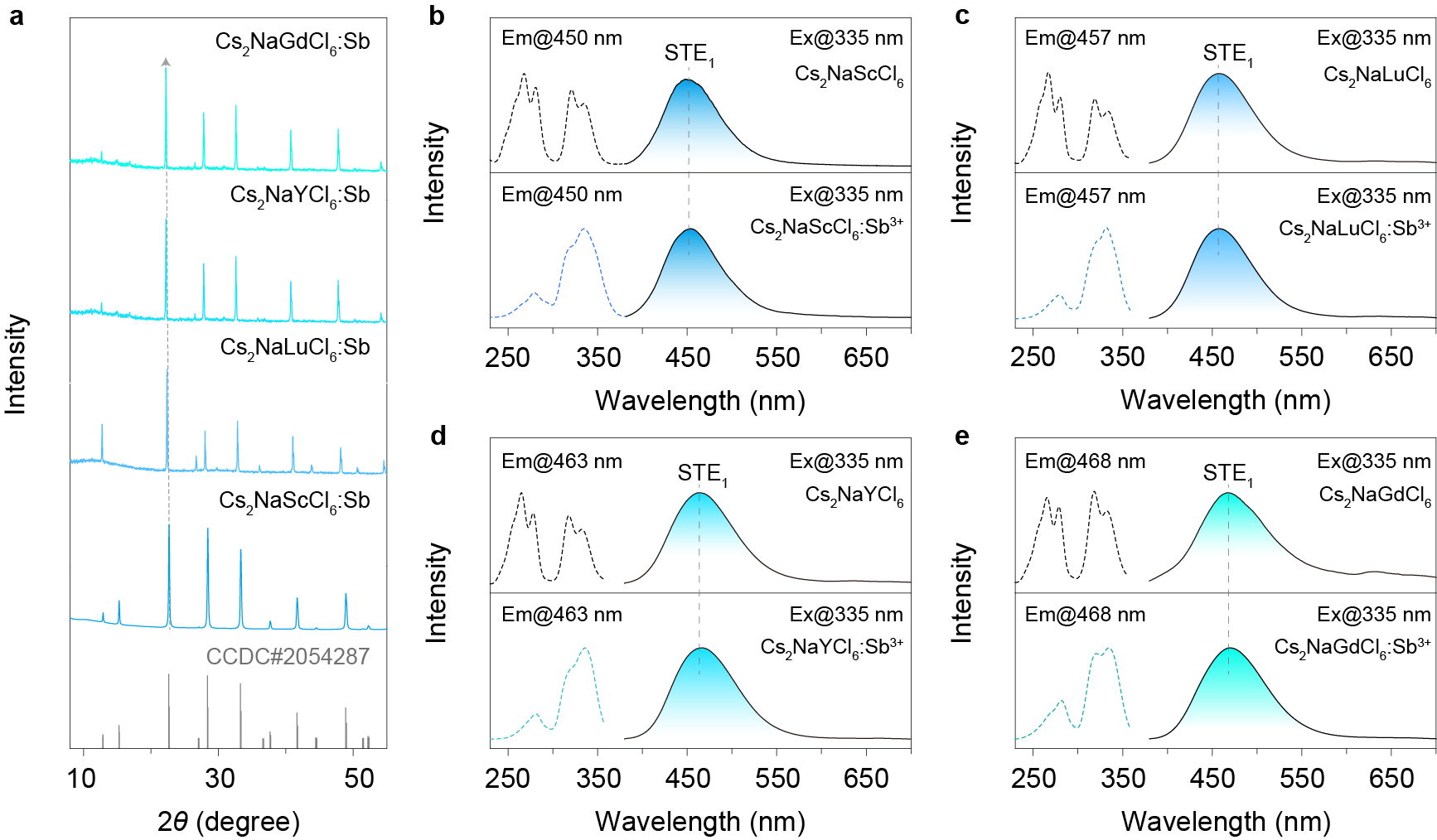
**

**Fig. S11 a,** XRD patterns and **b-e,** PLE (dotted lines) and PL (solid lines) spectra of bare Cs_2_NaRECl_6_ (RE = Sc, Lu, Y, and Gd, top panel) and Cs_2_NaRECl_6_:Sb^3+^ (0.75%) crystals (bottom panel). Through a modified hydrothermal synthesis, colorless transparent Cs_2_NaLuCl_6_, Cs_2_NaYCl_6_, Cs_2_NaGdCl_6_ crystals isostructural to Cs_2_NaScCl_6_ crystal were obtained. Under excitation at 335 nm, we detected the broadband emissions in bare Cs_2_NaRECl_6_ (RE = Sc, Lu, Y, and Gd) crystals that showed a close spectral resemblance to that of Sb^3+^-doped Cs_2_NaScCl_6_ counterparts except for the lower intensity. Note that the PL peak position gradually red-shifts from 450 to 468 nm as the ionic radii of rare-earth ions increase (Sc^3+^ < Lu^3+^< Y^3+^< Gd^3+^), which is typically observed for isolated Sb^3+^ ions with *ns^2^* configuration, explained in terms of reduced spin-orbit interaction by the Jahn-Teller effect^2^. The above results indicated that the high-energy broadband emissions in bare crystals are ascribed to STE recombination induced by Sb^3+^ impurities.

**
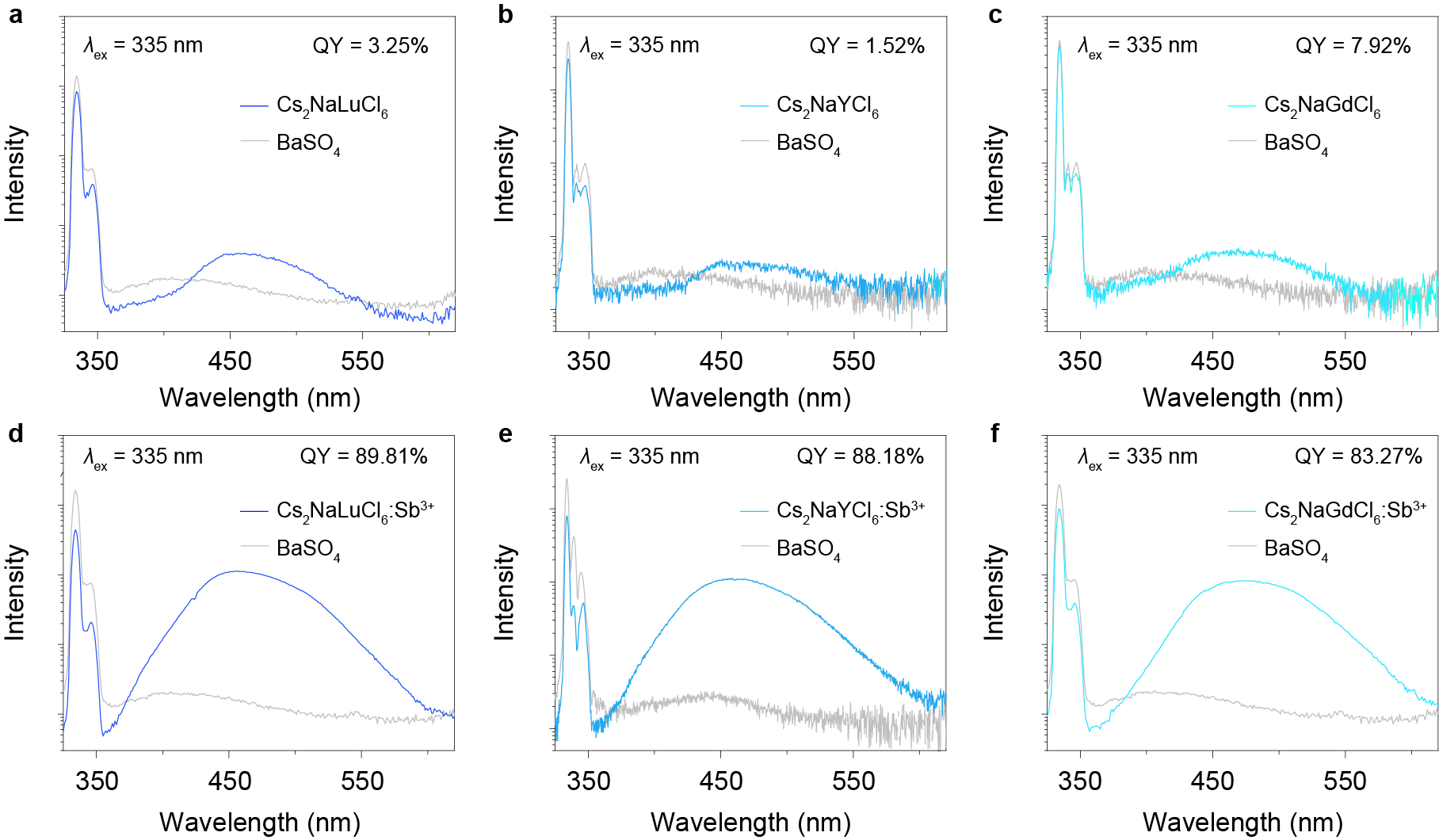
**

**Fig. S12** The PLQY measurements of bare and Sb^3+^ (0.75%)-doped Cs_2_NaRECl_6_ (RE = Lu, Y, and Gd) crystals.

**
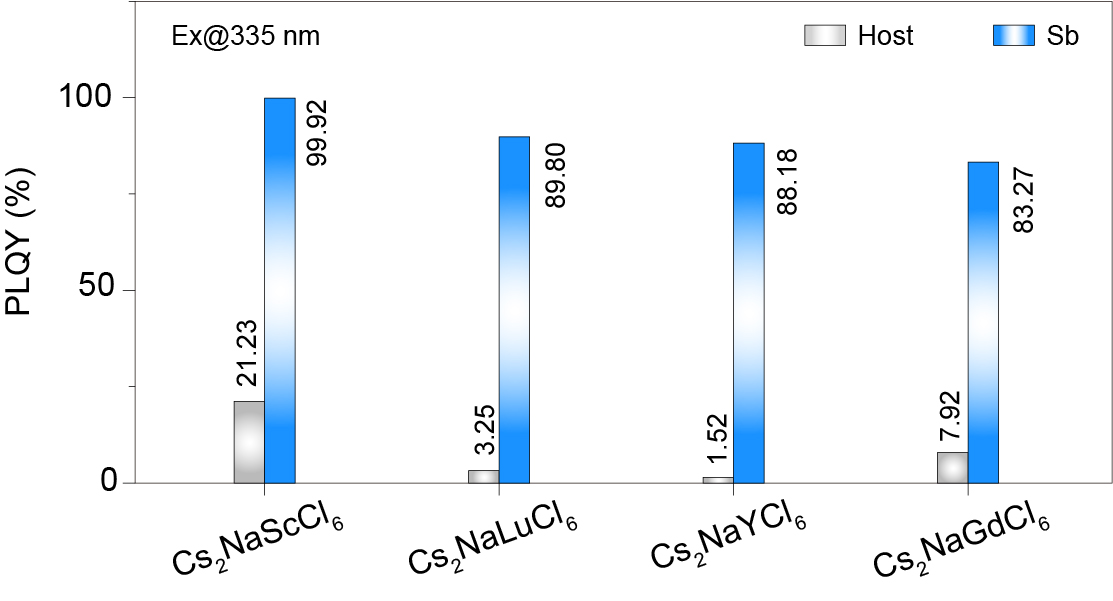
**

**Fig. S13** The PLQY values of bare and Sb^3+^ (0.75%)-doped Cs_2_NaRECl_6_ (RE = Sc, Lu, Y, and Gd) crystals. The PLQY of blue emission was substantially enhanced upon Sb^3+^ doping under 335 nm excitation. Among these crystals, Cs_2_NaScCl_6_:Sb^3+^ crystal shows the highest PLQY values of about 99.92% for high-energy STE emission.

**
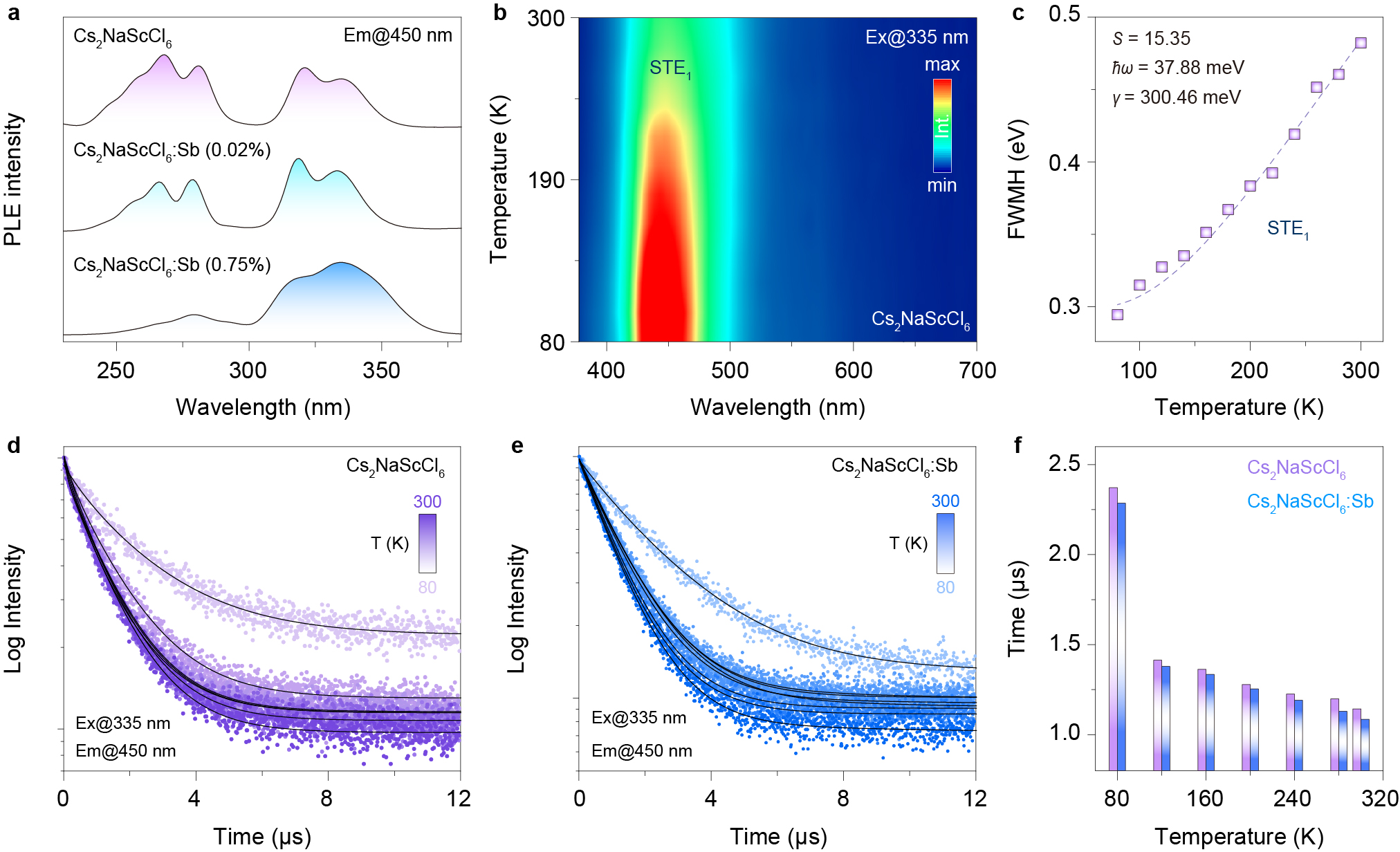
**

**Fig. S14 a**, Normalized PLE spectra of Cs_2_NaScCl_6_, Cs_2_NaScCl_6_:Sb^3+^ (0.02%) and Cs_2_NaScCl_6_:Sb^3+^ (0.75%) crystals by monitoring the emission at 450 nm. It can be found that the well-resolved fine structure of PLE peaks in bare crystals was nearly identical to that of the sample doped with a trace content of Sb^3+^ (0.02%). The noticeable broadening and restructuring of PLE bands was detected at a high doping level of Sb^3+^ due to the strengthened interactions among Sb^3+^ dopants, which has been widely reported in Sb^3+^-doped crystals^3^. **b,** Contour plot of the temperature-dependent PL spectra (80–300 K) of Cs_2_NaScCl_6_ crystal under 335 nm excitation. **c,** The obtained FWHM of blue emission *versus* the temperature. The experimental data were fitted to derive the optical phonon energy ($\text{ℏ}$*ω*_op_), Huang-Rhys factor (*S*), and Fröhlich coupling constant (*γ*_op_). These parameters were fitted to be $\text{ℏ}$*ω_op_* = 37.88 meV, *S* = 15.35, *γ*_op_ = 300.46 meV for blue emission, which were close to that of Sb^3+^-doped crystals. **d-e,** Temperature-dependent decay curves of blue emission at 450 nm by 335 nm excitation in Cs_2_NaScCl_6_ and Cs_2_NaScCl_6_:Sb^3+^ (0.75%) crystals, along with **f,** the calculated lifetimes as a function of temperature. As the temperature increased from 80 to 300 K, the calculated lifetimes of both crystals gradually decreased due to the promoted nonradiative relaxation. It is worth noting that the lifetimes of 450 nm emission in bare crystals were nearly identical to that of the Sb^3+^-doped counterparts. The above results confirmed the consistent origin of the high-energy emission before and after doping with Sb^3+^ ions, which are ascribed to STE recombination induced by Sb^3+^ ions rather than the perovskite host.


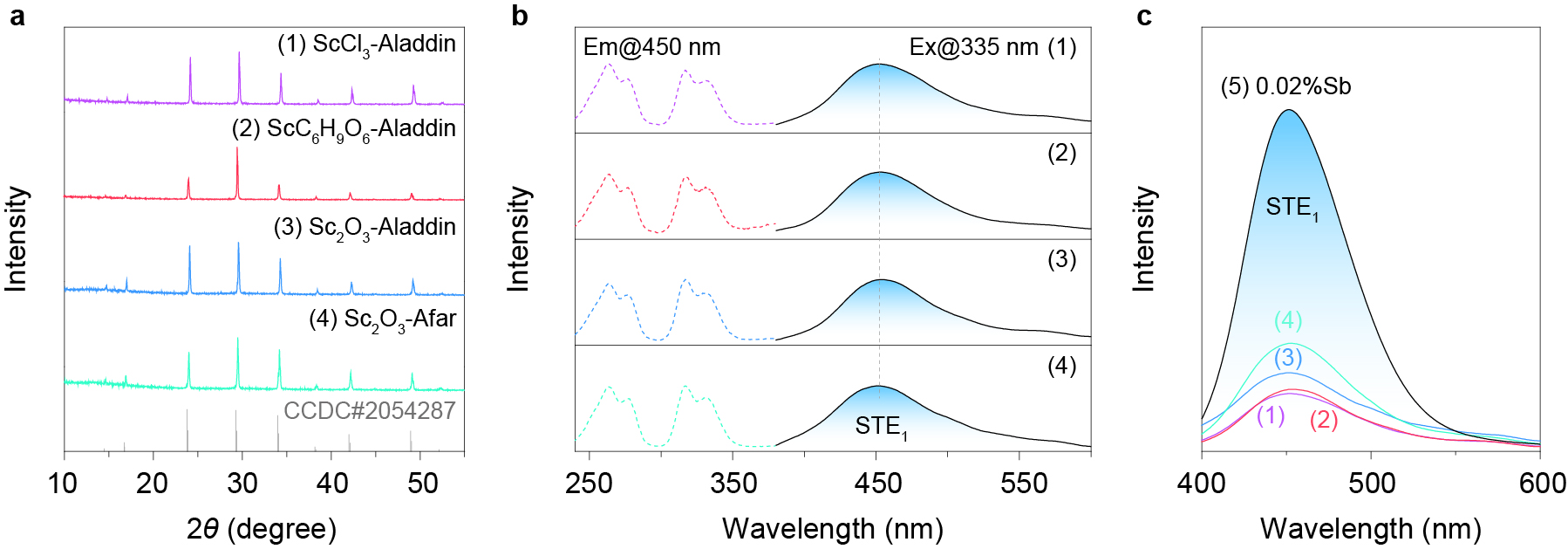


**Fig. S15 a**, XRD patterns and **b**, PLE (*λ*_em_ = 450 nm) and PL spectra (*λ*_ex_ = 335 nm) of bare Cs_2_NaScCl_6_ crystals prepared using reagents purchased from different suppliers as raw materials of Sc^3+^ element, including (1) ScCl_3_·6H_2_O-Aladdin, (2) ScC_6_H_9_O_6_·xH_2_O-Aladdin, (3) Sc_2_O_3_-Aladdin, and (4) Sc_2_O_3_-Alfa Aesar. **c**, PL spectra of (1-4) bare crystals and (5) Cs_2_NaScCl_6_:Sb^3+^ (0.02%) crystal under identical measurement conditions. Note that the emission intensity of bare crystals was enlarged by 10 times for better comparison. It is worth noting that we conducted several replicated experiments using high-purity unopened raw materials, new glassware, and fresh Teflon-lined containers. In all cases, the blue emission was consistently detectable.


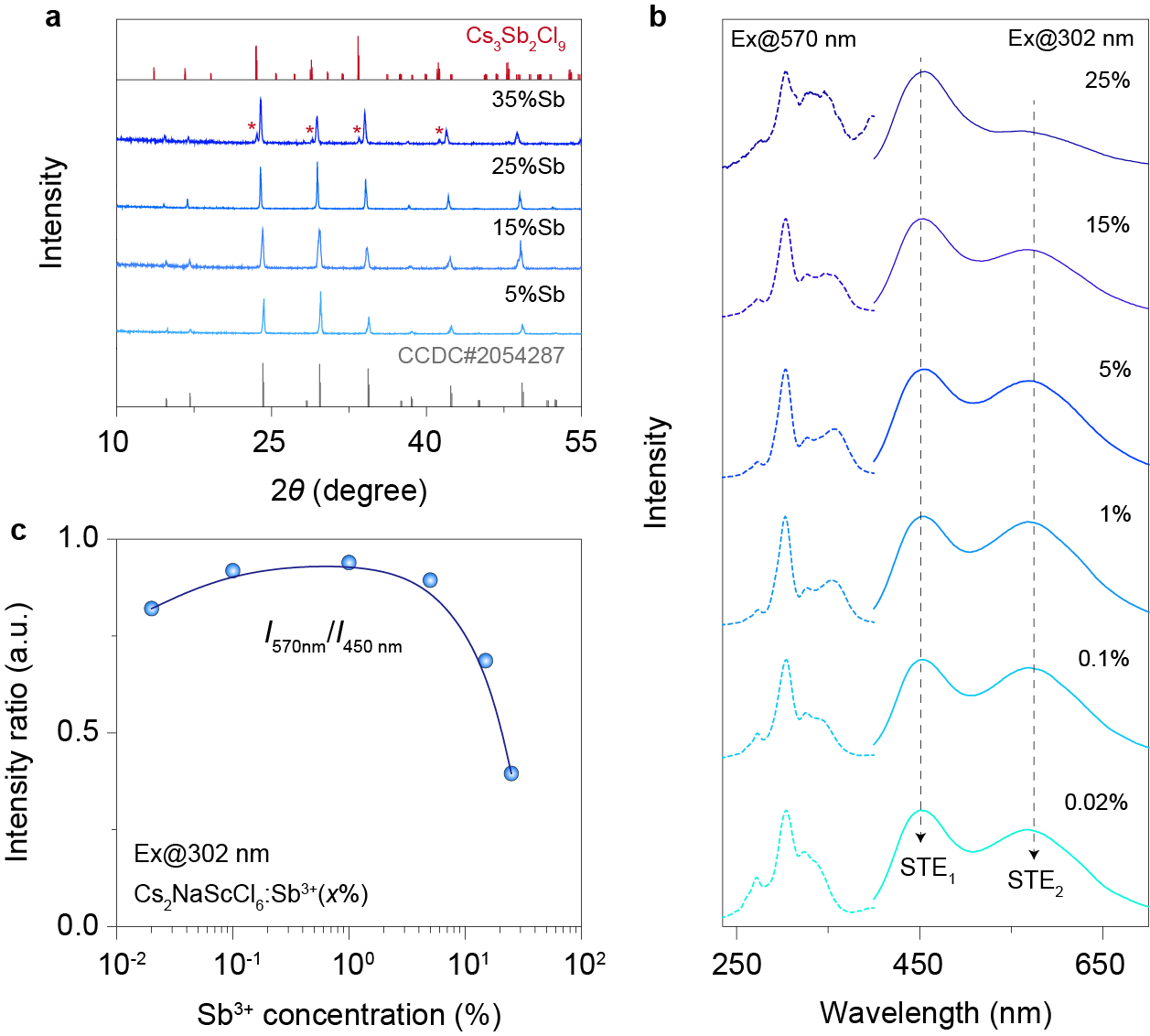


**Fig. S16 a**, XRD patterns of Cs_2_NaScCl_6_ crystals doped with high concentrations of Sb^3+^ (5–35%). Single-phase Cs_2_NaScCl_6_ crystals were obtained with high crystallinity when the dopant content was below 35%, while a further increase of Sb^3+^ resulted in impurity phase of Cs_3_Sb_2_Cl_9_. **b**, PLE (*λ*_em_ = 570 nm) and PL (*λ*_ex_ = 302 nm) spectra of Cs_2_NaScCl_6_:*x*Sb^3+^ (*x* = 0.02–25%) crystals. **c,** The calculated intensity ratio of 570 and 450 nm (*I*_570nm_/*I*_450nm_) as a function of Sb^3+^ concentration. It is worth noting that the emission band at 570 nm straightforwardly appeared after introducing a trace concentration of Sb^3+^ (0.02%), supporting the origin of yellow emission from the Sb^3+^-induced STE recombination. Note that the 570 nm emission was quickly quenched at high Sb^3+^ doping levels in comparison with the blue one.

**
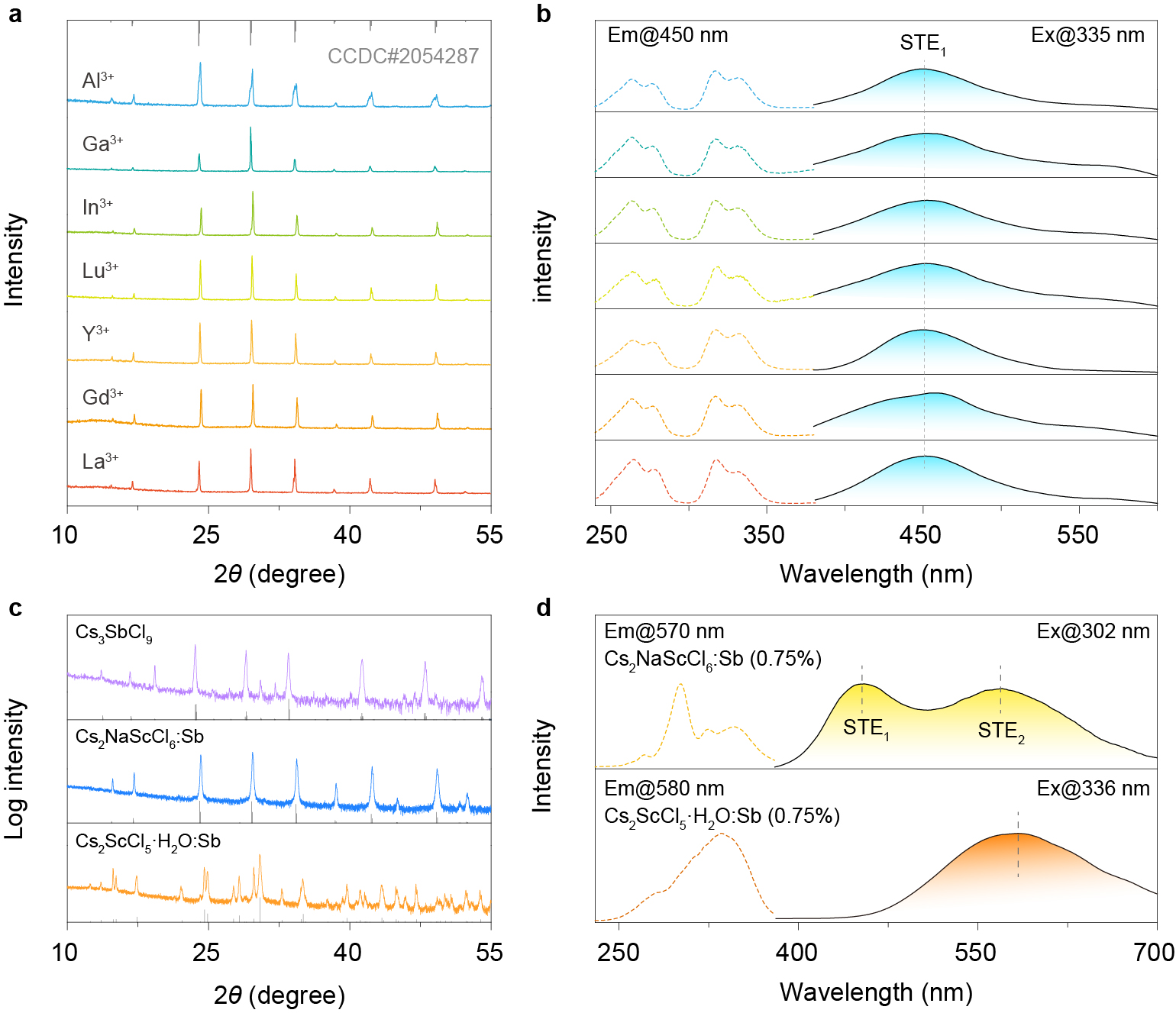
**

**Fig. S17 a**, XRD patterns and **b**, PLE (*λ*_em_ = 450 nm) and PL (*λ*_ex_ = 335 nm) spectra of Cs_2_NaScCl_6_ crystals doped with different trivalent ions (Al^3+^, Ga^3+^, In^3+^, Lu^3+^, Y^3+^, Gd^3+^, and La^3+^, 0.75%). Unlike Sb^3+^-doped crystal, these samples comprising trivalent ions with varied ionic radius (0.53–1.03 Å) only exhibited a single blue STE emission, indicating that the yellow emission was unlikely ascribed to doping-induced defect states. **c,** XRD patterns of as-prepared Cs_2_NaScCl_6_:Sb^3+^ (0.75%), Cs_2_ScCl_5_·H_2_O:Sb^3+^ (0.75%) and Cs_3_Sb_2_Cl_9_ crystals. Note that the impurity phases of Cs_2_ScCl_5_·H_2_O and Cs_3_Sb_2_Cl_9_ were hardly detected in as-prepared Cs_2_NaScCl_6_ crystal doped with 0.75%Sb^3+^. **d**, PLE and PL spectra of Cs_2_NaScCl_6_:Sb^3+^ and Cs_2_ScCl_5_·H_2_O:Sb^3+^ (0.75%) crystals. Note that no emission was detected in Cs_3_Sb_2_Cl_9_ crystal. There were obvious differences in PLE and PL profiles between Cs_2_NaScCl_6_:Sb^3+^ and Cs_2_ScCl_5_·H_2_O:Sb^3+^ crystals. The above results indicated that the origin of yellow emission is unlikely credited to impurities related to Cs_2_ScCl_5_·H_2_O and Cs_3_Sb_2_Cl_9_.


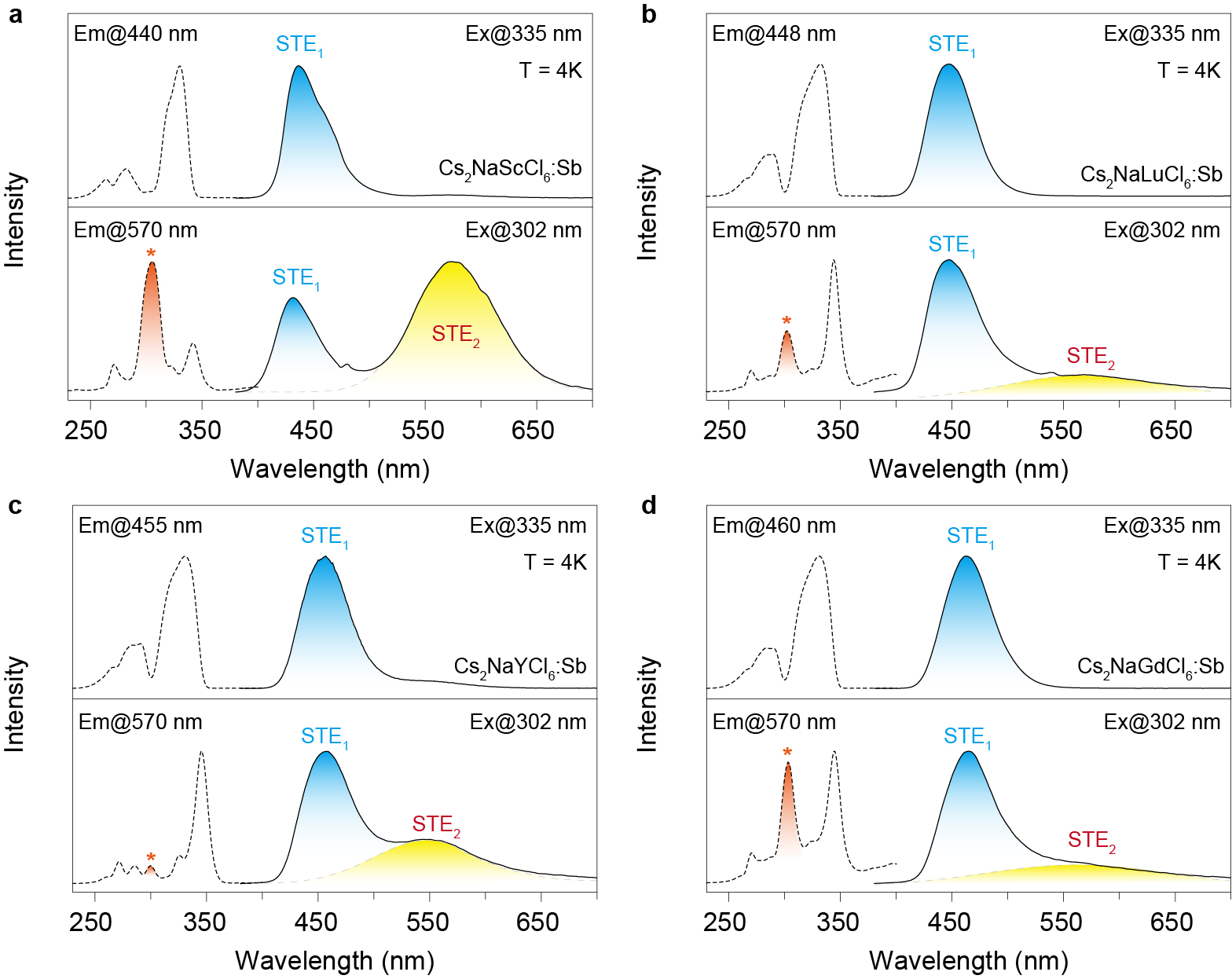


**Fig. S18** PLE and PL spectra of Sb^3+^ (0.75%)-doped **a,** Cs_2_NaScCl_6_, **b,** Cs_2_NaLuCl_6_, **c,** Cs_2_NaYCl_6_, and **d,** Cs_2_NaGdCl_6_ crystals at 4 K. Under excitation at 335 nm, all samples exhibited a single STE emission, in consistency with PL data at RT. By contrast, two STE emissions were detected at low temperatures under 302 nm excitation. However, the low-energy STE emission in Cs_2_NaRECl_6_:Sb^3+^ (RE = Lu, Y, Gd) was much weaker than that in Cs_2_NaScCl_6_:Sb^3+^, manifesting as a shoulder to the high-energy blue emission.


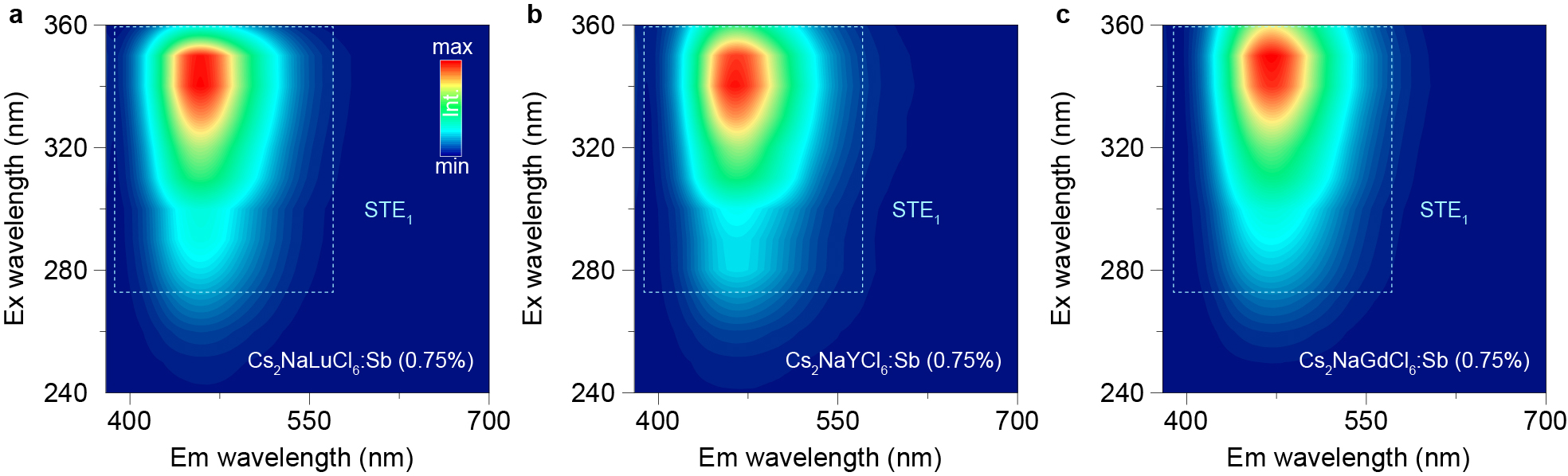


**Fig. S19** Contour plot of the excitation-wavelength-resolved PL spectra for Sb^3+^ (0.75%)-doped **a,** Cs_2_NaLuCl_6_, **b,** Cs_2_NaYCl_6_, and **c,** Cs_2_NaGdCl_6_ crystals at RT. Unlike Cs_2_NaScCl_6_:Sb^3+^ crystal, these rare-earth halide double-perovskite crystals exhibited only a single luminescent center at RT.

**
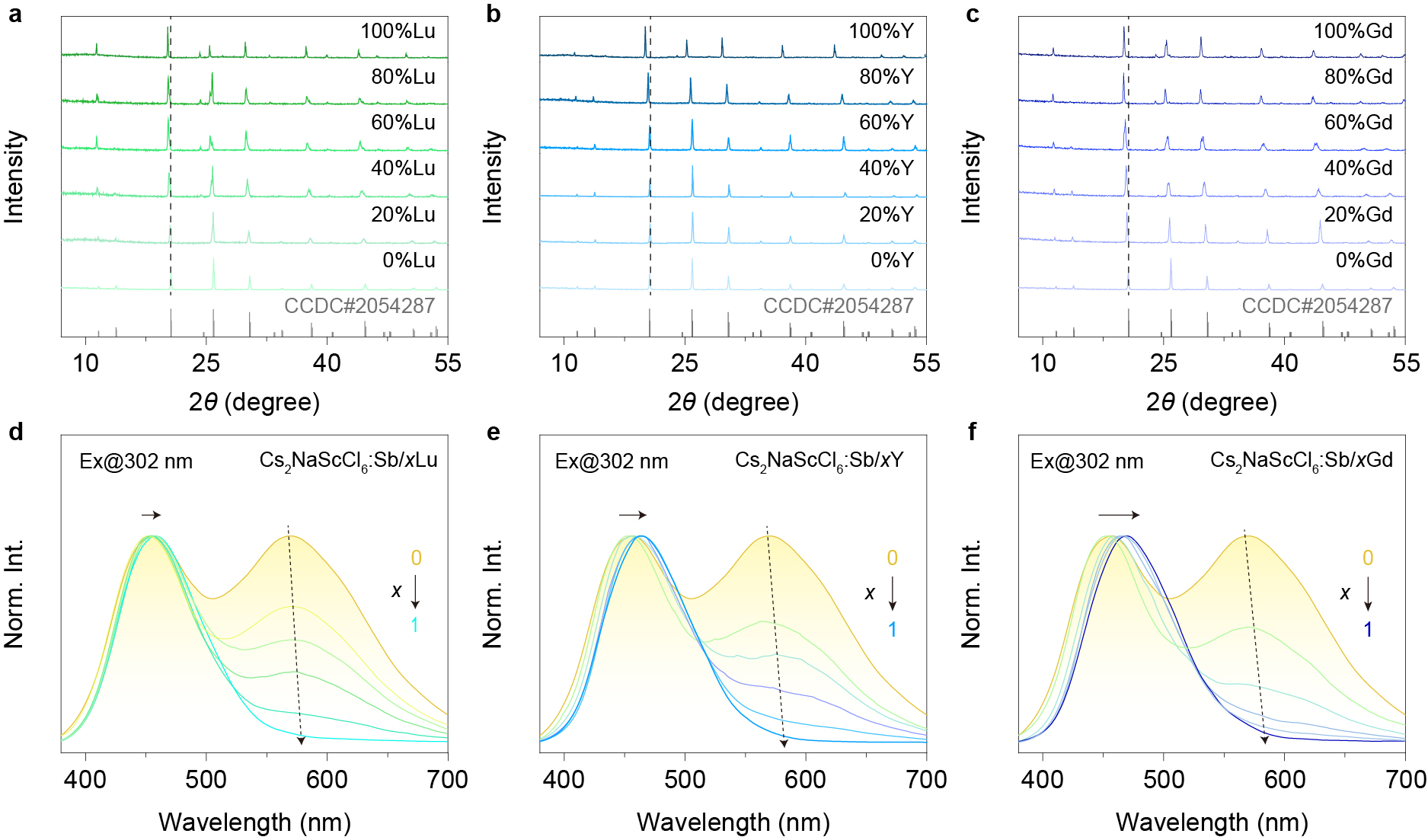
**

**Fig. S20 a-c,** XRD patterns and **d-f,** normalized PL spectra of Cs_2_NaScCl_6_:Sb^3+^/RE^3+^ (RE = Lu, Y, and Gd, 0.75%/0–100%) crystals under 302 nm excitation. We found a gradual shift of the main diffraction peaks towards lower angles with increasing the dopant concentration, indicating the successful substitution of RE^3+^ ions for smaller Sc^3+^ without inducing secondary phases. Meanwhile, the yellow emission at 570 nm sharply decreased with increasing the dopant concentration, accomplished by the progressive red-shift of the peak position. It is worth noting that the introduction of rare-earth ion with a larger ionic radius induced a faster reduction of the 570 nm emission. We thus reasoned that the smallest ionic radius of Sc^3+^ among rare-earth ions might favor the Sb^3+^-related yellow STE emission in halide double-perovskite crystals.

**
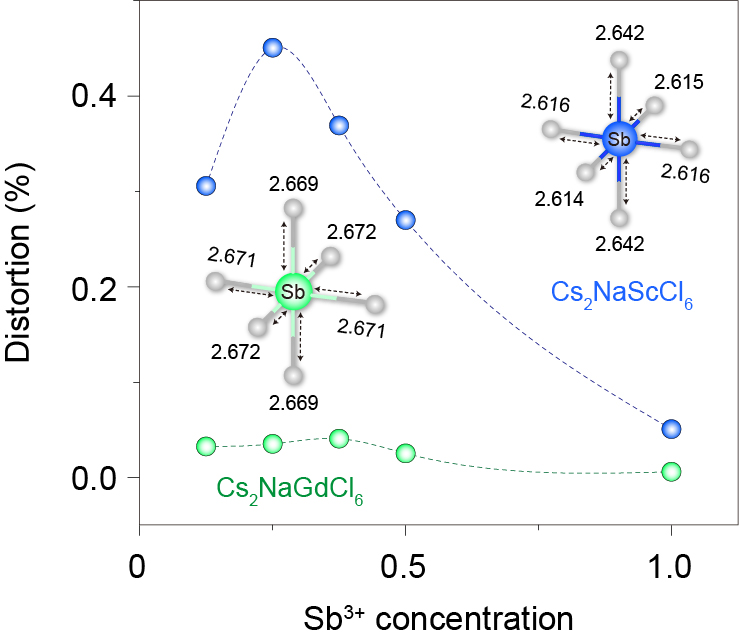
**

**Fig. S21** The distortion (*D_dis_*) of [SbCl_6_]^3-^ units derived from the optimized crystal structure of Cs_2_NaScCl_6_ and Cs_2_NaGdCl_6_ as a function of Sb^3+^ doping concentration. Insets show the optimized [SbCl_6_]^3-^ octahedron of Cs_2_NaSc_0.75_Sb_0.25_Cl_6_ and Cs_2_NaGd_0.75_Sb_0.25_Cl_6_. Note that the distortion index (*D_dis_*) is employed to quantify the structural distortion of [SbCl_6_]^3-^ unit, which can be defined as:^4^

$\text{D}_{\text{dis}}\text{=}\frac{\text{1}}{\text{n}}\sum_{\text{i=}\text{1}}^{\text{n}} \frac{\left| \text{d}_{\text{i}}\text{-}\text{d}_{\text{av}} \right|}{\text{d}_{\text{av}}}$ (S1)

where *n* denotes the coordination number, *d_i_* denotes the bond length between the central atom (Sb) and the *i*th coordinating atom (Cl), and *d_av_* represents the average bond length. Notably, the theoretical *D_dis_* value for [SbCl_6_]^3-^ unit was appreciably higher in Cs_2_NaScCl_6_ than Cs_2_NaGdCl_6_ crystal upon Sb^3+^ doping.

**­­
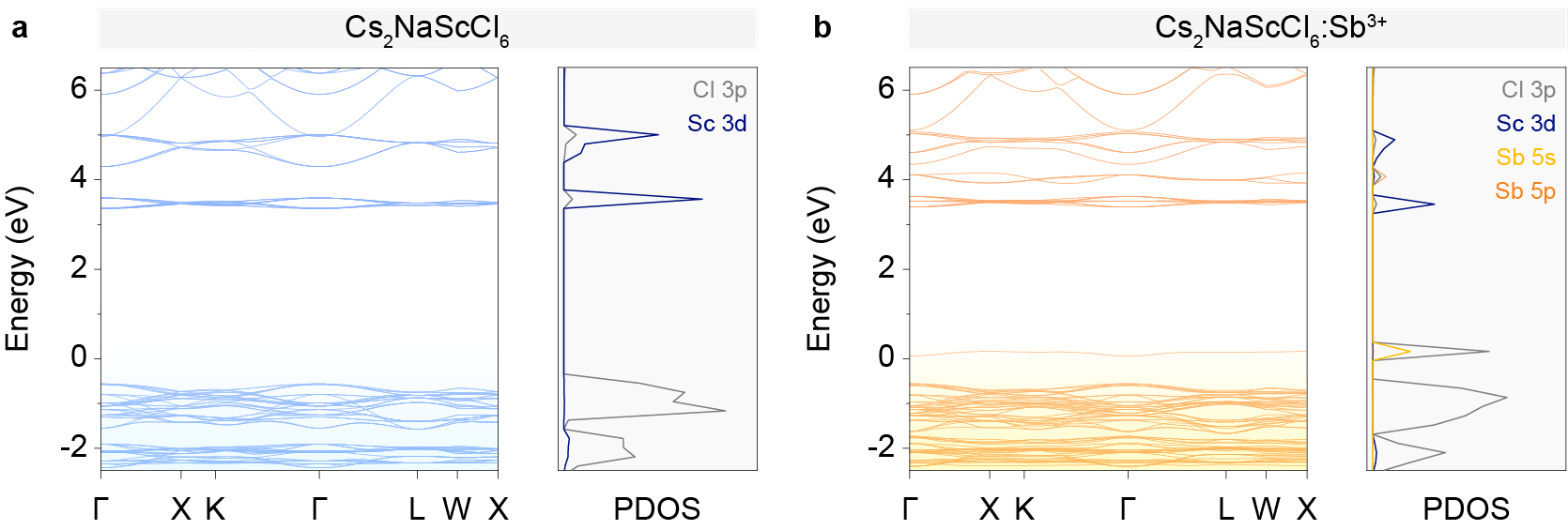
**

**Fig. S22** The calculated electronic band structures and PDOS of **a,** Cs_2_NaScCl_6_ and **b,** Cs_2_NaScCl_6_:Sb^3+^. In specific, Cs_2_NaScCl_6_ exhibits a direct bandgap at the *Γ* point with an energy of 3.91 eV, and the conduction band minimum (CBM) and valence band maximum (VBM) mainly derived from Sc 3d and Cl 3p orbitals, respectively. Upon Sb^3+^ doping, the formation of Cl 3p-hybridized Sb^3+^ ground state 5s above the original VBM results in a reduction of the band gap to 3.24 eV.

**
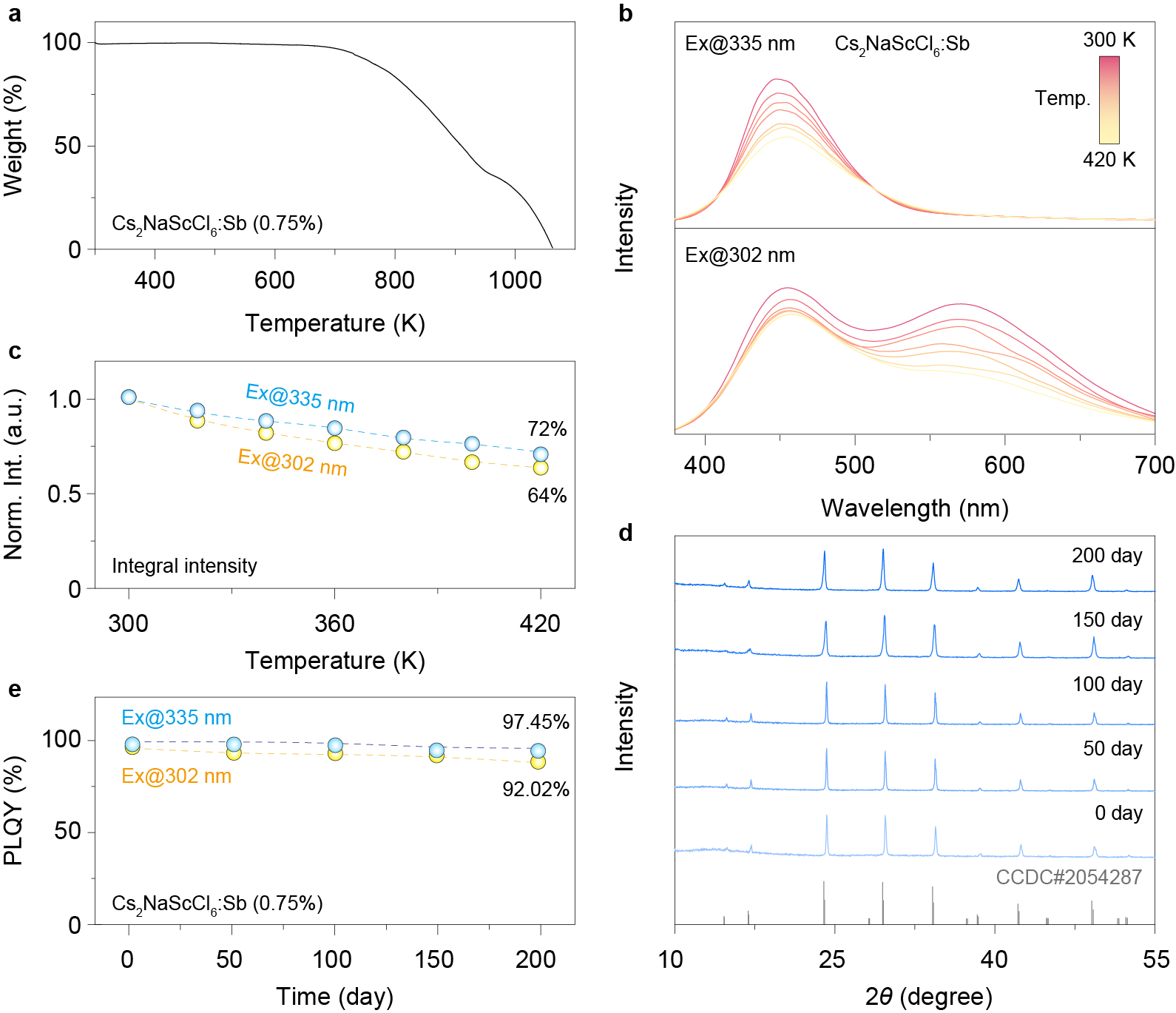
**

**Fig. S23 a,** TGA curve of Cs_2_NaScCl_6_:Sb^3+^ (0.75%) crystal. **b,** Temperature-dependent PL spectra of Cs_2_NaScCl_6_:Sb^3+^ crystal under 335 and 302 nm excitation, respectively. **c,** Normalized integral PL intensity as a function of temperature. **d,** XRD patterns and **e**, PLQY values of as-prepared Cs_2_NaScCl_6_:Sb^3+^ crystal under 335 and 302 nm excitation after 200 days of exposure to ambient air. The results demonstrated the excellent thermal and air stability of as-prepared Cs_2_NaScCl_6_:Sb^3+^ single crystals.

**
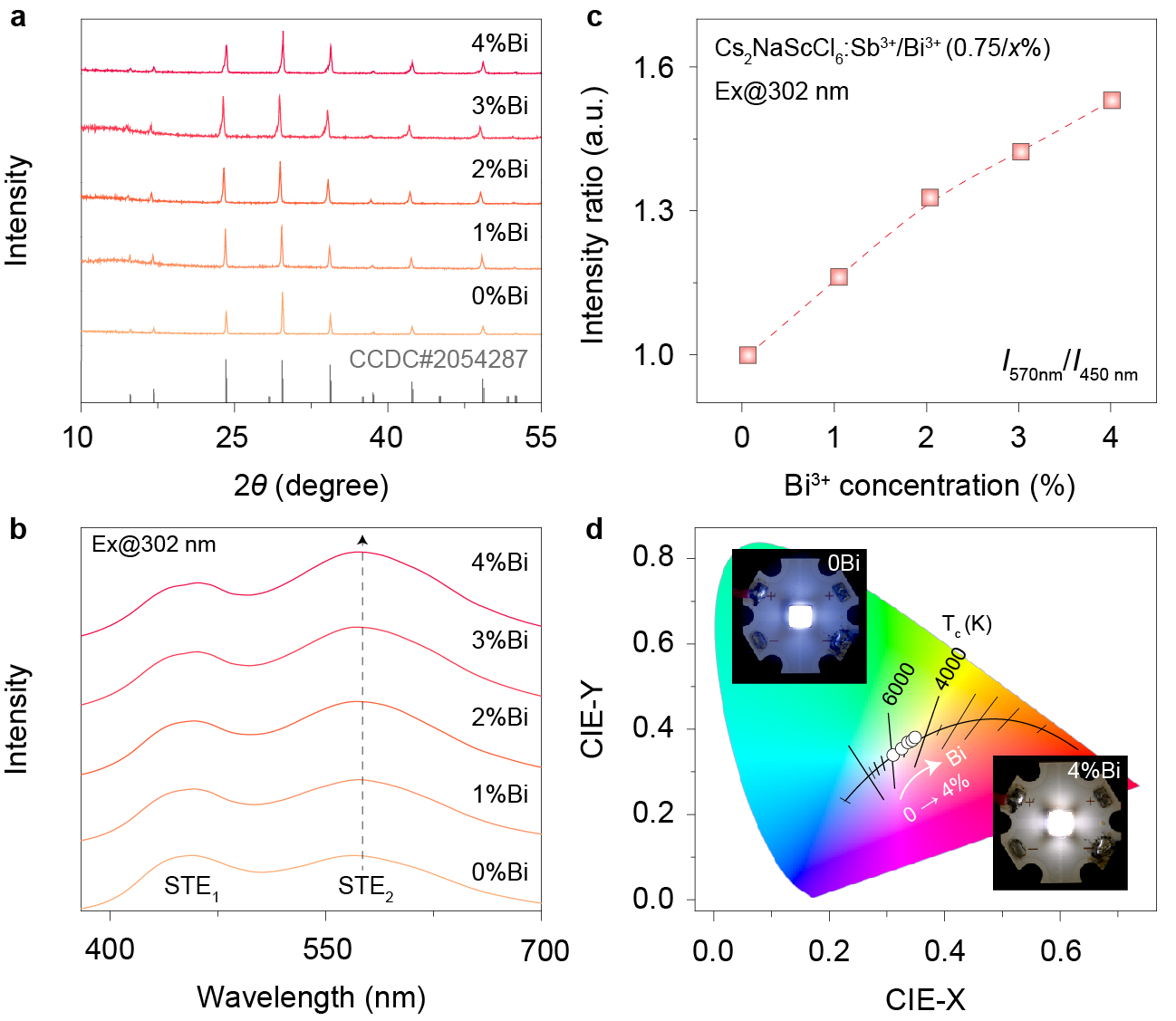
**

**Fig. S24 a,** XRD patterns and **b,** PLE (*λ*_em_ = 570 nm) and PL (*λ*_ex_ = 302 nm) spectra of Cs_2_NaScCl_6_:Sb^3+^/*y*Bi^3+^ (0.75/0–4%) crystals. **c,** The calculated intensity ratio of 570 and 450 nm (*I*_570nm_/*I*_450nm_) as a function of Bi^3+^ concentration. **d,** CIE chromaticity diagram of Cs_2_NaScCl_6_:Sb^3+^/*y*Bi^3+^ (0.75/0–4%) crystals, along with the luminescence images of two WLED devices fabricated by coating Cs_2_NaScCl_6_:Sb^3+^ (0.75%) and Cs_2_NaScCl_6_:Sb^3+^/Bi^3+^ (0.75/4%) crystals on a 310 nm LED chip. With increasing Bi^3+^ concentration, the yellow PL component gradually dominated over the blue, with no obvious changes in PLE bands. Previous work revealed that the introduction of Bi^3+^ ion can induce Jahn-Teller distortion of [SbCl_6_]^3-^ octahedron to improve the yellow STE intensity^5^. As a result, the correlated color temperature (CCT) of as-fabricated WLED devices can be fine-tuned from 6000 to 4000 K by increasing the dopant concentration of Bi^3+^ ions.


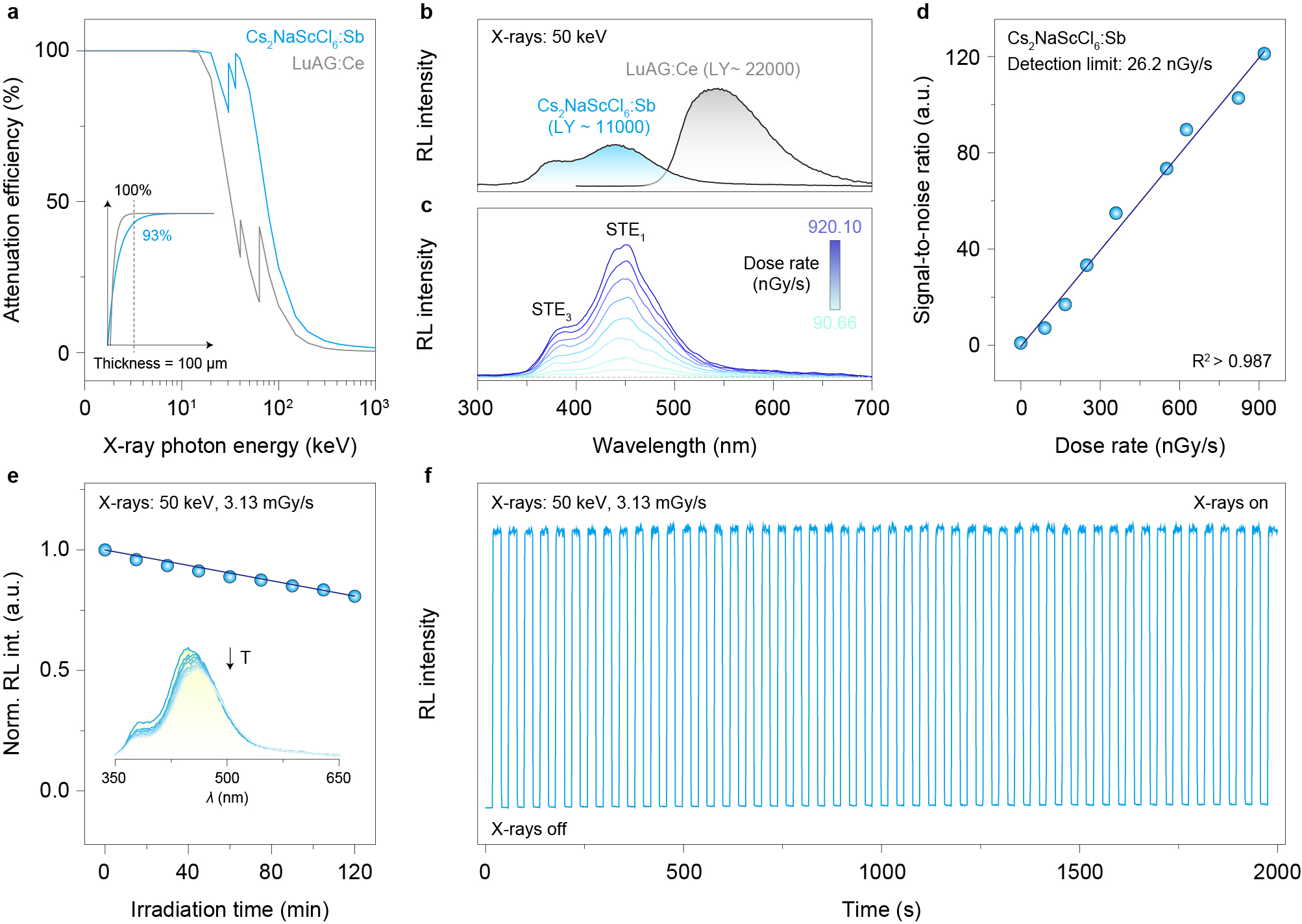


**Fig. S25 a,** X-ray attenuation efficiency of Cs_2_NaScCl_6_:Sb^3+^ (0.75%) and commercial LuAG:Ce^3+^ scintillator as a function of X-ray photon energy at a thickness of 100 μm. Inset shows the enlarged detail of X-ray attenuation efficiency at the thickness of 100 µm used for the estimation of light yield (LY). Note that the X-ray absorption ability of Cs_2_NaScCl_6_:Sb^3+^ was comparable to that of mainstream scintillators. **b,** RL spectra of Cs_2_NaScCl_6_:Sb^3+^ and LuAG:Ce^3+^ under X-ray excitation at a dose rate of 4.5 mGy·s^-1^. Under X-ray excitation, we detected two main STE emissions related to [ScCl_6_]^3-^ (STE_3_) and [SbCl_6_]^3-^ (STE_1_) in Cs_2_NaScCl_6_:Sb^3+^ crystal. To obtain the LY, the powder samples were compressed into scintillator wafers by a hydraulic press (15 MPa for 5 min), which were placed at the same position in the integrating sphere to measure the RL spectra. Based on the reported method, the LY value of Cs_2_NaScCl_6_:Sb^3+^ was determined to be around 11,000 photons·MeV^-1^ using the commercial LuAG:Ce^3+^ scintillator as a reference (~ 22,000 photons·MeV^-1^)^6^. **c,** RL spectra and **d,** the calculated signal-noise ratio of the crystal as a function of dose rate by increasing X-ray tube current from 5–70 μA. We found a linear response of RL intensity to X-ray dose rate, and the detection limit was measured to be 26.2 nGy·s^-1^ as the signal-noise ratio is 3, which is much lower than the standard dose of 5.5 μGy·s^-1^ for X-ray examination^7^. **e,** RL intensity as a function of X-ray irradiation time or **f,** on-off cycles at a dose rate of 3.31 mGy·s^-1^. Under continuous or repeated X-ray irradiation at a high dose rate, Cs_2_NaScCl_6_:Sb^3+^ crystal exhibited excellent irradiation stability with no noticeable decline in RL intensity. Interestingly, RL intensity gradually increased after ceasing X-ray excitation during the on-off cyclic measurement, inspiring us to further explore the X-ray-excited PersL of Cs_2_NaScCl_6_:Sb^3+^ crystal.

**
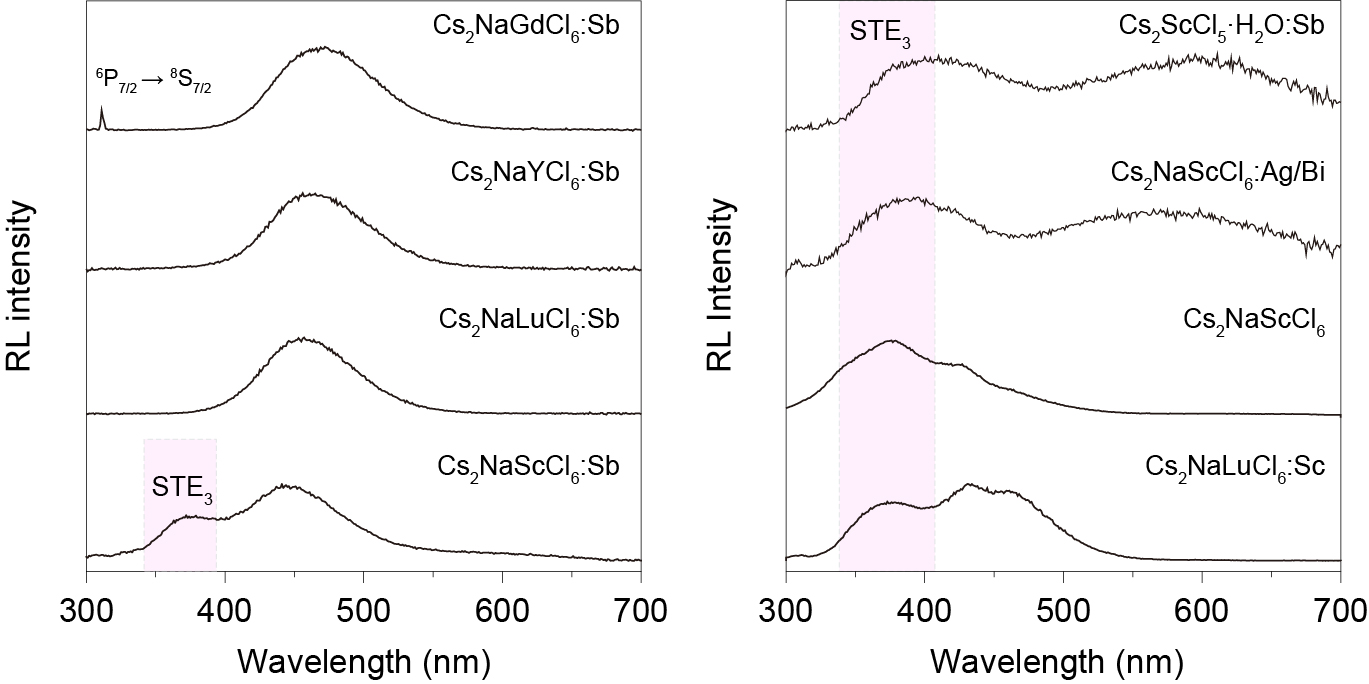
**

**Fig. S26** Comparison of RL spectra of Cs_2_NaRECl_6_:Sb^3+^ (0.75%, RE = Sc, Lu, Y, Gd), Cs_2_NaScCl_6_, Cs_2_NaLuCl_6_:Sc^3+^ (1%), Cs_2_NaScCl_6_:Ag^+^/Bi^3+^ (1%/1%), and Cs_2_ScCl_5_·H_2_O:Sb^3+^ (0.75%) crystals. Notably, the high-energy broadband emission was only detected in Sc^3+^-contained halide perovskites upon X-ray excitation, possibly attributing to the Sc^3+^-related STE recombination.

**
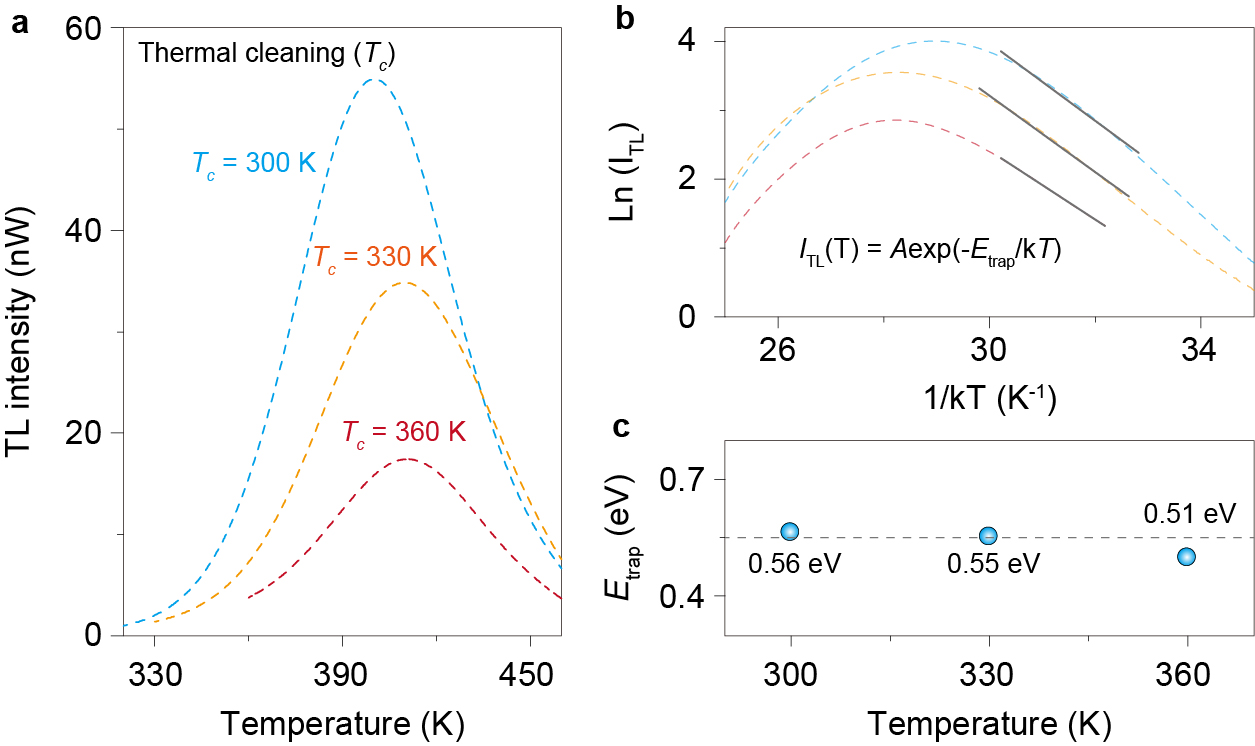
**

**Fig. S27 a,** TL spectra of Cs_2_NaScCl_6_:Sb^3+^ (0.75%) crystal recorded using the thermal cleaning method. The sample was first heated to designated temperatures (300, 330, and 360 K) after X-ray charging at RT for 15 minutes. Following thermal cleaning for 1 minute, the TL curves were recorded by continued heating to 460 K at a constant heating rate of 2.5 K·s^-1^. **b,** Arrhenius plot [Ln(I_TL_) versus kT^-1^] of the TL curves and **c,** the estimated trap depths based on the initial-rise analysis. The trap depths were fitted to be around 0.51–0.56 eV with a narrow distribution, suggesting the presence of a single PersL-active trap state in Cs_2_NaScCl_6_:Sb^3+^ crystal.

**
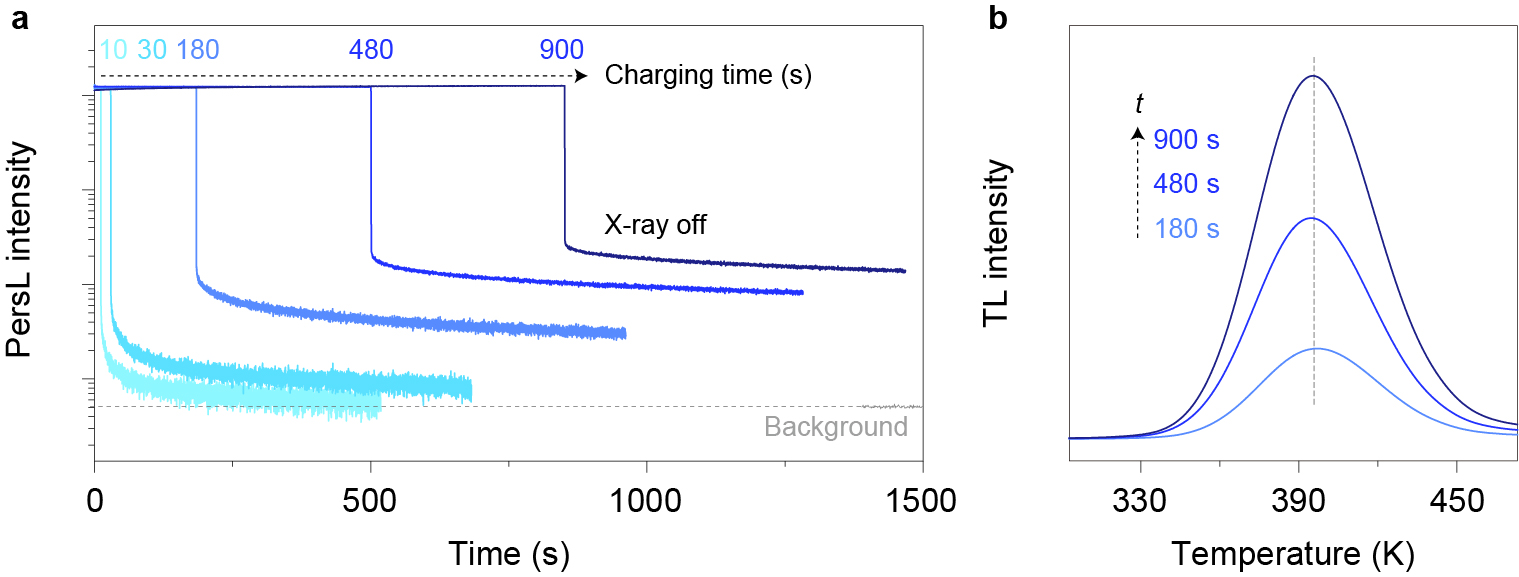
**

**Fig. S28 a,** PersL decay profiles and **b,** TL spectra of Cs_2_NaScCl_6_:Sb^3+^ crystal as a function of charging time. It can be clearly found that the PersL performance (intensity and duration) was gradually improved by prolonging the X-ray irradiation time, in consistency with the TL intensity. Note that the TL signals were too weak to be accurately detected for short charging times (< 30 s).


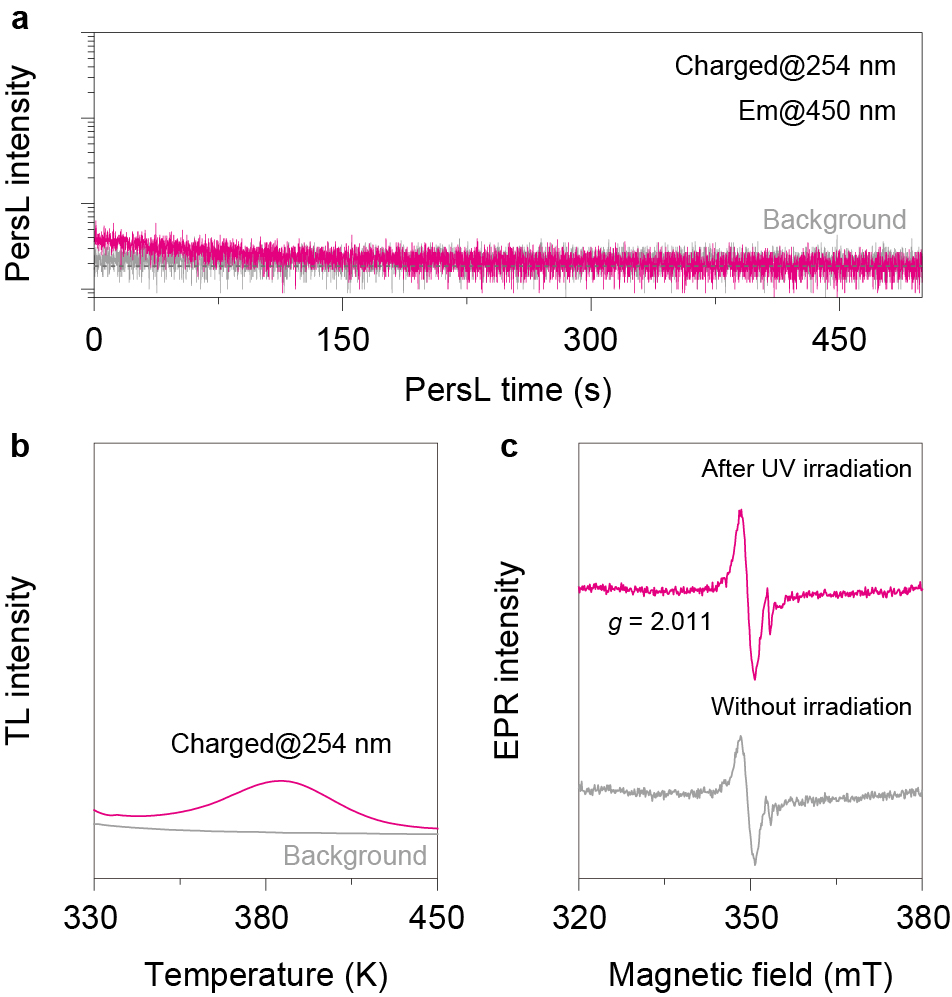


**Fig. S29 a,** PersL decay profile and **b,** TL spectra of Cs_2_NaScCl_6_:Sb^3+^ crystal after the cessation of UV light. The crystal was pre-charged using a 254 nm UV lamp (12 W) for 3 minutes before the measurement. Note that UV light charging hardly resulted in observable PersL, possibly due to its relatively low photon energy. **c,** EPR spectra of as-prepared Cs_2_NaScCl_6_:Sb^3+^ crystal recorded before and after 254 nm irradiation at RT. It can be found that the intensity of the original EPR signal in the crystal hardly changed upon UV charging, indicating that effective trap-filling requires high-energy irradiation.


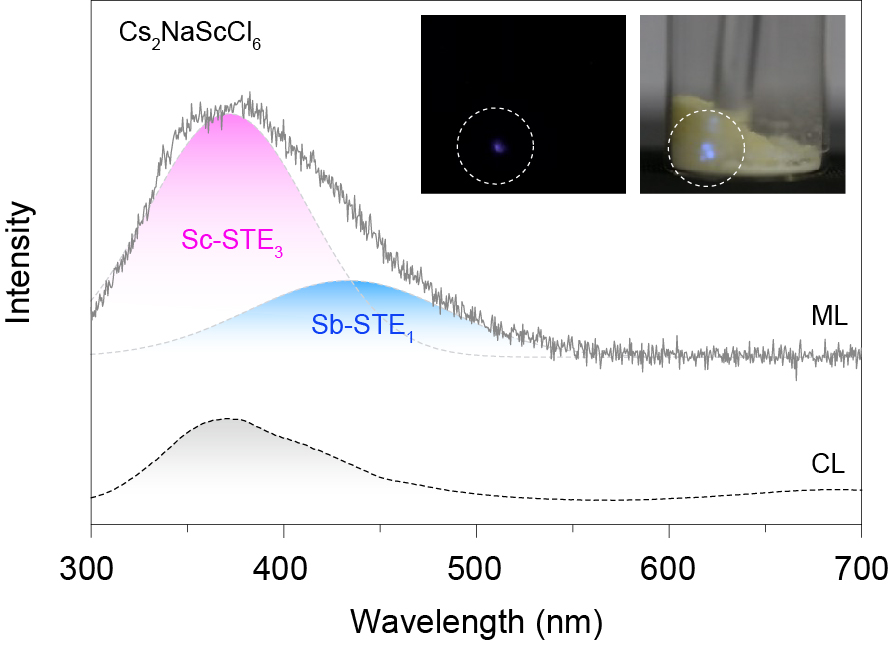


**Fig. S30** ML and CL spectra of bare Cs_2_NaScCl_6_ crystal. Insets show the ML photographs of crystals in ambient light or dark under continuous grinding with a glass rod. Notably, the ML spectrum of bare crystal was mainly composed of violet broadband emissions corresponding to Sc^3+^-related STE recombination, in close resemblance to the X-ray-excited RL spectrum. The above results further confirmed that the Sc^3+^-related STE emission necessitates high-energy excitation.

**
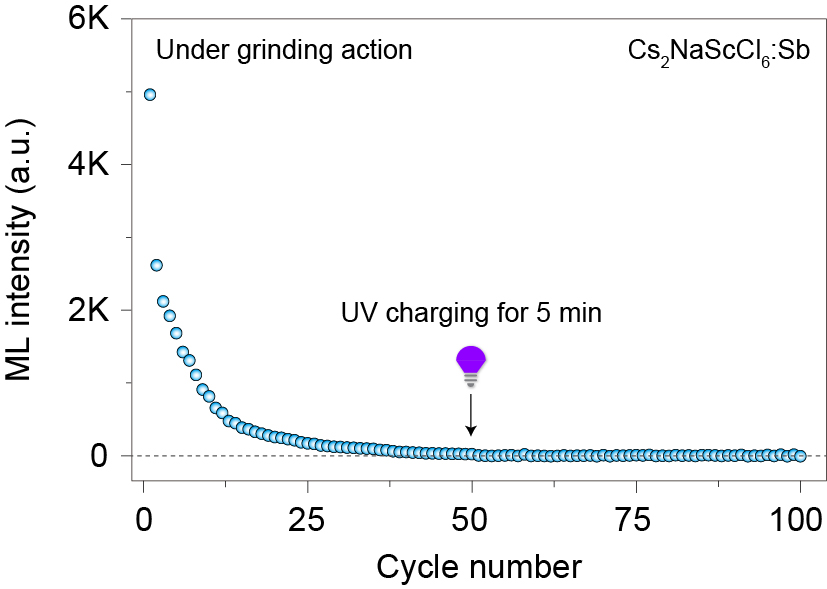
**

**Fig. S31** The cyclic stability of ML intensity in Cs_2_NaScCl_6_:Sb^3+^ (0.75%) crystals in the dark under continuous grinding. The ML intensity gradually diminished as the single crystals were fragmented into powders, which could hardly recover by UV charging.

**
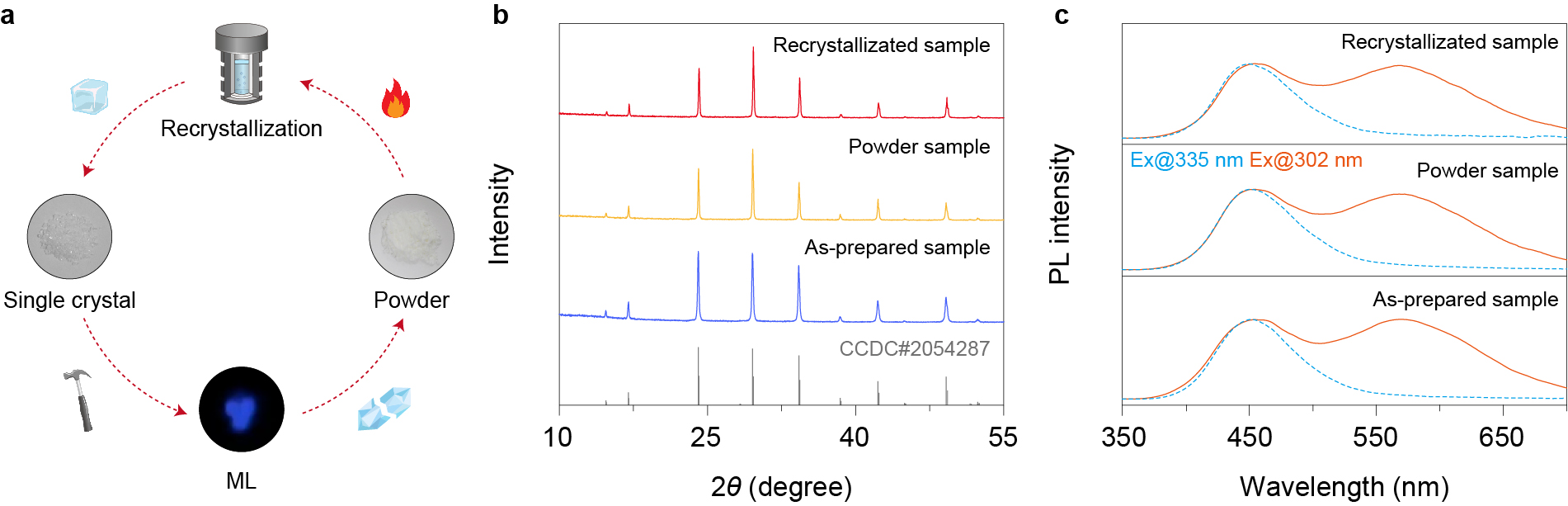
**

**Fig. S32 a,** Schematic illustration of reproducible ML in Cs_2_NaScCl_6_:Sb^3+^ crystal, along with the ML photographs. **b,** XRD patterns of as-prepared crystal, powder, and recrystallized crystal of Cs_2_NaScCl_6_:Sb^3+^ (0.75%). **c,** PL spectra of as-prepared crystal, powder, and recrystallized crystal under 302 and 335 nm excitation. Through a simple recrystallization process, the fine powder sample can be transformed into single crystals under repeated hydrothermal treatment. It is worth noting that the evolution of sample form hardly affected its crystal phase and PL property. More importantly, the ML emission disappeared as the single crystals were fragmented into fine powders, which was completely recovered by the recrystallization process. Results indicated the reproducible nature of ML performance in Cs_2_NaScCl_6_:Sb^3+^ crystal.

**
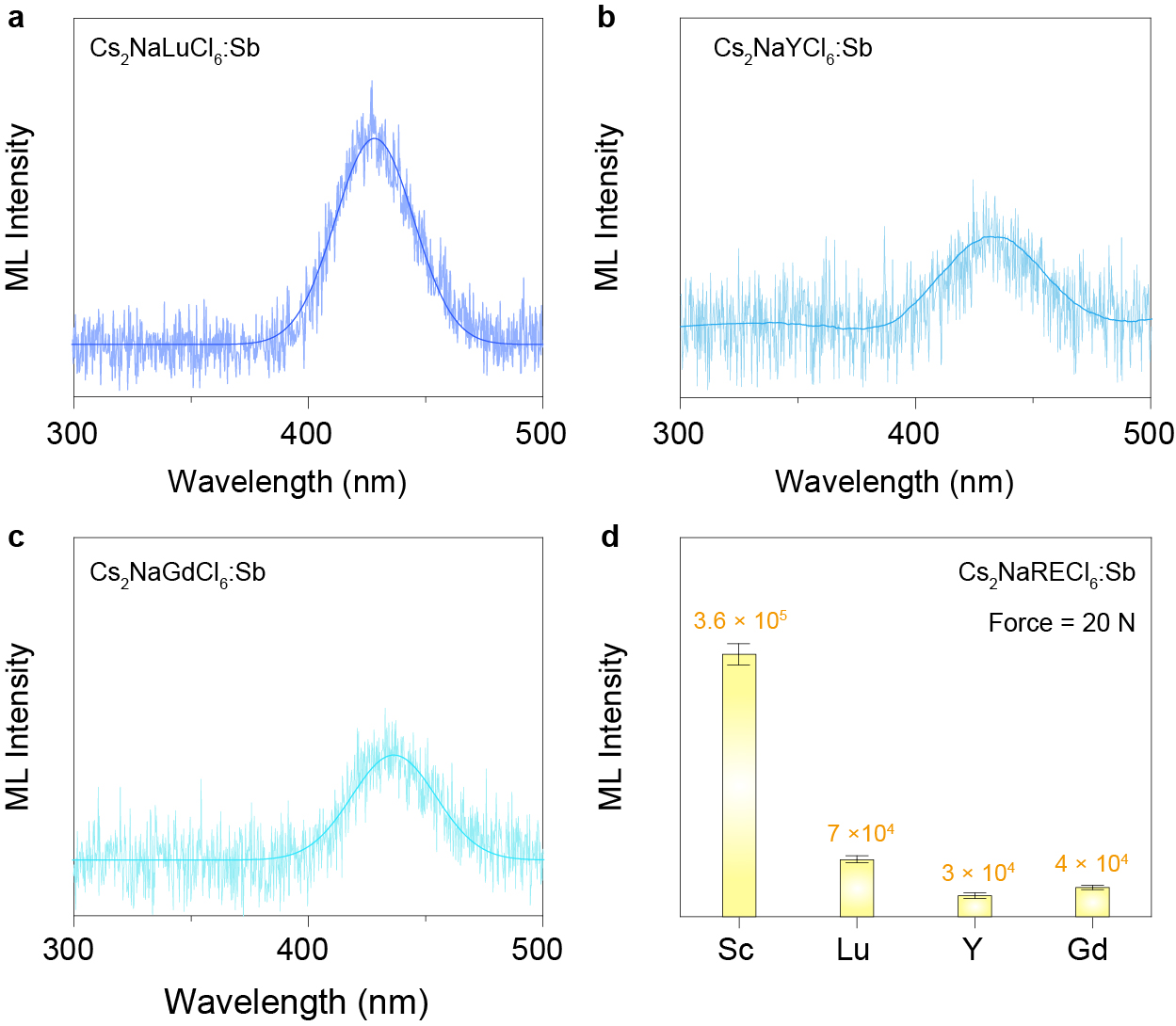
**

**Fig. S33 a-c,** ML spectra of Sb^3+^-doped Cs_2_NaLuCl_6_, Cs_2_NaYCl_6_, and Cs_2_NaGdCl_6_ crystals with grain sizes of around 2 mm under mechanical excitation at 20 N. **d,** Comparison of integral ML intensity among Cs_2_NaRECl_6_:Sb^3+^ (0.75%) crystals under identical measurement conditions. Compared with Cs_2_NaScCl_6_:Sb^3+^ crystal, a single STE ML was detected with much lower intensity in these Sb^3+^-doped rare-earth halide double-perovskites.


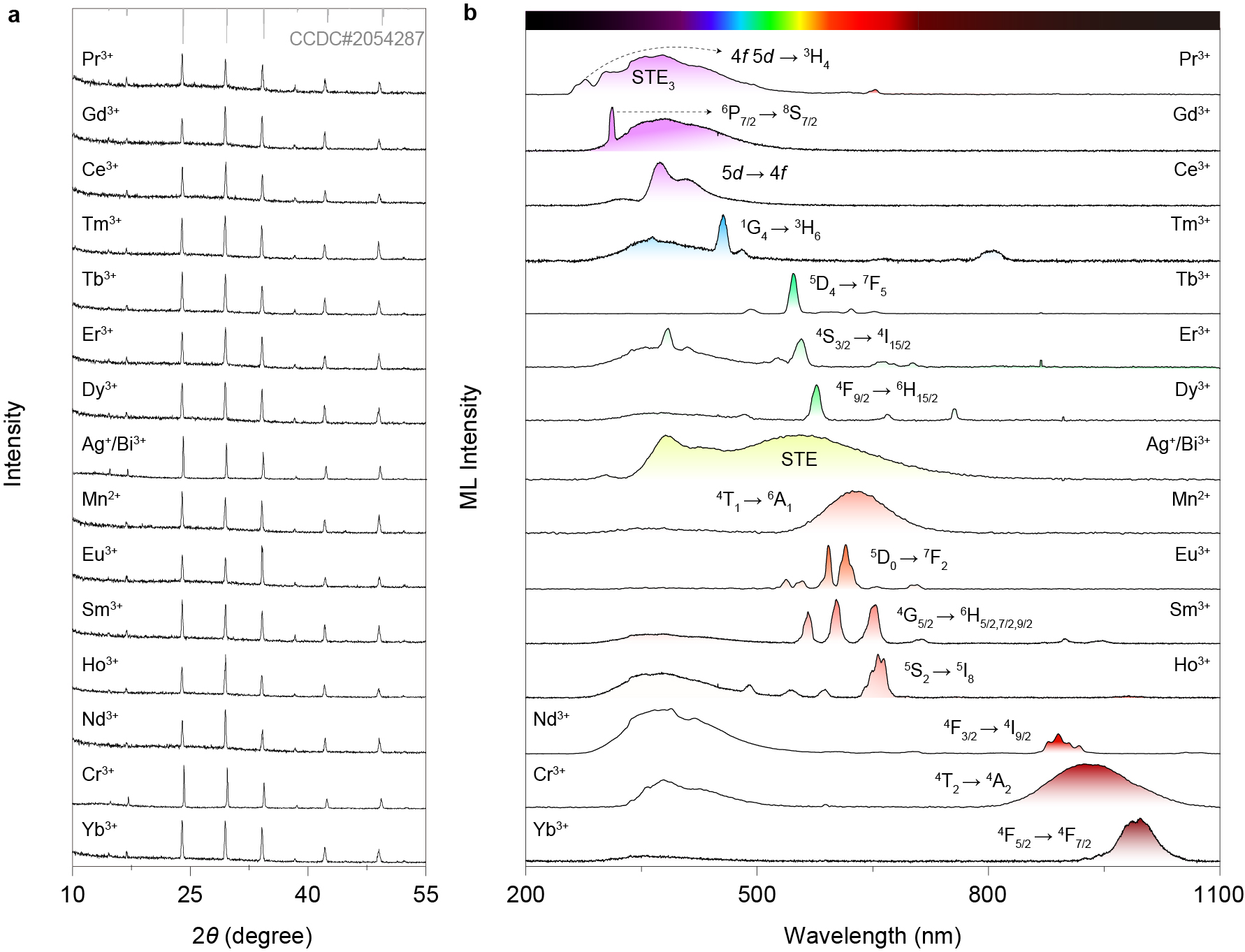


**Fig. S34 a,** XRD patterns and **b,** ML spectra of Cs_2_NaScCl_6_ crystals doped with various activators (RE^3+^, Mn^2+^, Cr^3+^, and Ag^+^/Bi^3+^) in the range of 200–1100 nm. Owing to the high doping capacity of Sc^3+^ site, we successfully incorporated a family of trivalent rare-earth (RE = Yb^3+^, Tm^3+^, Er^3+^, Ho^3+^, Dy^3+^, Tb^3+^, Gd^3+^, Eu^3+^, Sm^3+^, Nd^3+^, Pr^3+^, and Ce^3+^), transition metal (TM = Mn^2+^ and Cr^3+^), and main group metal ions (Ag^+^/Bi^3+^) into Cs_2_NaScCl_6_ crystals as ML centers for flexible multicolor tuning. Remarkably, bright ML emissions from distinct electronic transitions, including ns^2^-nsnp, 3*d*-3*d*, 5*d*-4*f*, 4*f*-4*f*, and exciton recombination, were detected in a single host crystal spanning a broad spectral range from ultraviolet C (UVC) to NIR, which is inaccessible to the existing ML materials. It is worth noting that the obtained UVC emission at around 275 nm of Pr^3+^ ion is one of the shortest ML wavelengths.


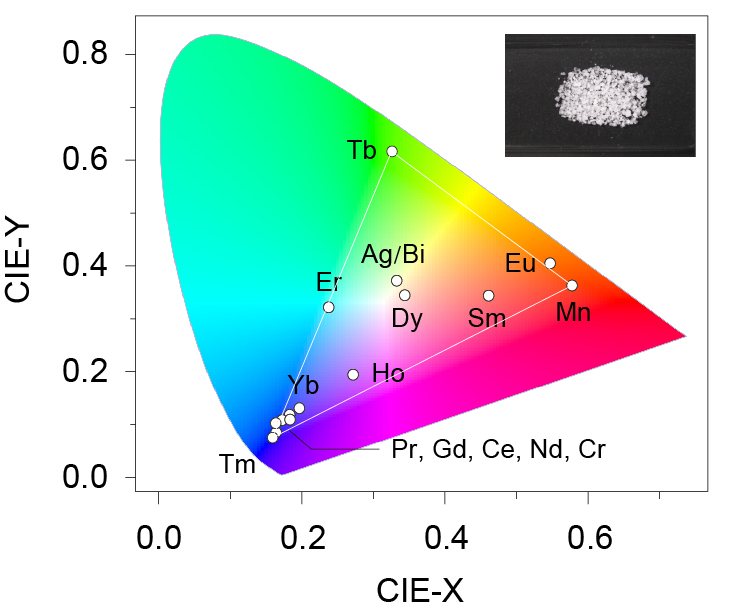


**Fig. S35** CIE chromaticity diagram of Cs_2_NaScCl_6_ singles crystals doped with various activators (RE^3+^, Mn^2+^, Cr^3+^, Sb^3+^, and Ag^+^/Bi^3+^). Inset shows the photograph of single crystals sealed in a flexible PET film. Notably, full-spectrum bright ML emissions covering a wide color gamut were realized within Cs_2_NaScCl_6_ single crystals.


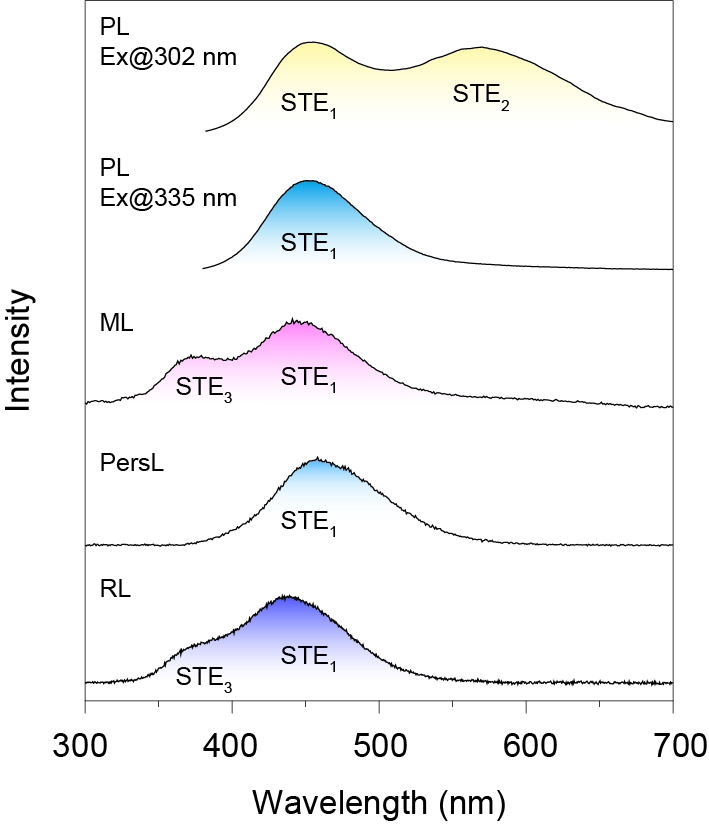


**Fig. S36** Excitation-mode-selective multiexcitonic emissions of Cs_2_NaScCl_6_:Sb^3+^ crystal. It is clearly found that Sb^3+^-induced yellow STE_2_ emission was only detectable under the excitation at 302 nm, while intense blue STE_1_ emission appeared under all forms of excitation. In addition, high-energy excitation, such as X-rays and electron beams (Cathode rays), is required for Sc^3+^-related violet STE_3_ emission.

**III. Supplementary Tables**

**Table S1** Calculated formation energy (*E_form_*) in Cs_2_NaScCl_6_:Sb^3+^ crystal under different occupational schemes.

| **Crystal phase** | **Occupied sites** | ***E_form_* (eV)** |
| --- | --- | --- |
| Cs_2_NaScCl_6_ | Sb → Cs | -6.74 |
|  | Sb → Na | -5.74 |
|  | Sb → Sc | -10.63 |

**Table S2** Rietveld refinement and the calculated crystallographic data for Cs_2_NaScCl_6_:Sb^3+^ crystals.

| **Sb^3+^ content** | **0** | **0.25%** | **0.50%** | **0.75%** | **1%** | **1.25%** |
| --- | --- | --- | --- | --- | --- | --- |
| **Space group** | *Fm-3m* | | | | | |
| ***Z*** | 4 | | | | | |
| ***a* =*b* =*c* (Å)** | 10.4898 | 10.4901 | 10.4904 | 10.4906 | 10.4917 | 10.4922 |
| ***V* (Å^3^)** | 1154.278 | 1154.364 | 1154.472 | 1154.541 | 1154.883 | 1155.055 |
| ***R*_wp_** | 8.7% | 9.0% | 8.8% | 8.8% | 9.0% | 8.1% |
| ***R*_p_** | 6.66% | 6.69% | 6.48% | 6.57% | 6.80% | 6.09% |
| ***χ*^2^** | 1.21 | 1.35 | 1.29 | 1.31 | 1.40 | 1.47 |

**Table S3** Comparison of multimode luminescence among the reported Sb^3+^-doped metal halides.

| **Materials** | **Ex** | **Em** | **FWHM** | **PLQY** | **PersL** | **ML** | **Ref.** |
| --- | --- | --- | --- | --- | --- | --- | --- |
| (C_4_N_2_H_12_)_3_(NH_4_)_4_Cd_4_Cl_18_:Sb^3+^ | 355 | 565 | 144 | 89.4% | - | - | 8 |
| ATPP_2_SnCl_6_:Sb^3+^ | 315 | 640 | 120 | 73.8% | - | - | 9 |
| (CH_3_NH_3_)_4_InCl_7_:Sb^3+^ | 326 | 612 | 180 | 84% | - | - | 10 |
| (NH_4_)_x_(OH_3_)_3-x_InCl_6_:Sb^3+^ | 280/290 | 550/630 | 18.7/19.3 | 86% | - | - | 11 |
| Cs_2_InCl_5_·H_2_O:Sb^3+^ | 340 | 580 | 135 | 95.5% | - | - | 12 |
| Rb_3_InCl_6_:Sb^3+^ | ~ 320 | 479 | ~ 120 | 95% | - | - | 13 |
| Rb_3_YbCl_6_:Sb^3+^ | 365 | 533 | 120 | 66.9% | - | - | 14 |
| Cs_2_ZnCl_4_:Sb^3+^ | 316 | 745 | 175 | 69.98% | - | - | 15 |
| Cs_2_SnCl_6_:Sb^3+^ | 365 | 601 | 101 | 15.9% | - | - | 16 |
| Cs_2_ZrCl_6_:Sb^3+^ | 315/490 | 490/600 | / | 43.1% | - | - | 17 |
| Cs_2_AgInCl_6_:Sb^3+^ | 370 | 660 | 195 | 32% | - | - | 18 |
| Cs_2_KInCl_6_:Sb^3+^ | 320 | 510 | 80 | 6.22% | - | - | 19 |
| Cs_2_NaInCl_6_:Sb^3+^, Bi^3+^ | 302 | 450/570 | 77/156 | 77% | - | - | 20 |
| Cs_2_NaHoCl_6_:Sb^3+^ | 334 | 462 | 77 | 97% | - | - | 21 |
| Cs_2_NaYCl_6_:Sb^3+^ | 320 | 461 | 82 | 82.5% | - | - | 22 |
| Cs_2_NaLuCl_6_:Sb^3+^ | 338 | 454 | 74 | 68% | - | - | 23 |
| CsCdCl_3_:Sb^3+^ | 353 | 524 | 109 | 59.6% | 1.38 h | - | 24 |
| Cs_3_Cd_2_Cl_7_:Sb^3+^ | 254 | 517 | 100 | 2.54% | 2 s | - | 25 |
| Cs_2_NaScCl_6_:Sb^3+^ | 302/335 | 450/570 | 72/210 | 97.45/99.92% | > 9 h | + | This work |
| “+” or “-” denote the sample with or without PersL/ML property | | | | | | | |

**Table S4** ICP-MS results of as-prepared Cs_2_NaScCl_6_ and Cs_2_NaScCl_6_:Sb^3+^ crystals.

| **Sample** | **Actual content of Sb^3+^ (mg·Kg^-1^)** |
| --- | --- |
| Lu_2_O_3_ (Lu_2_O_3_-Afar) | 1.3248 |
| Cs_2_NaLuCl_6_ (Lu_2_O_3_-Afar) | 2.1569 |
| YCl_3_ (YCl_3_-Aladdin) | 0.5604 |
| Cs_2_NaYCl_6_ (YCl_3_-Aladdin) | 0.8609 |
| GdCl_3_ (GdCl_3_-Aladdin) | 2.9026 |
| Cs_2_NaGdCl_6_ (GdCl_3_-Aladdin) | 3.5301 |
| Sc_2_O_3_ (Sc_2_O_3_-Afar) | 91.6525 |
| Cs_2_NaScCl_6_ (ScCl_3_-Aladdin) | 4.5156 |
| Cs_2_NaScCl_6_ (ScC_6_H_9_O_6_-Aladdin) | 15.3086 |
| Cs_2_NaScCl_6_ (Sc_2_O_3_-Aladdin) | 26.0236 |
| Cs_2_NaScCl_6_ (Sc_2_O_3_-Afar) | 70.9525 |
| Cs_2_NaScCl_6_:Sb (0.02%, Sc_2_O_3_-Afar) | 1011.6458 |

**IV. Supplementary Reference**

1. Li, G. Q. et al. Regulating Exciton De-Trapping of Te^4+^-Doped Zero-Dimensional Scandium-Halide Perovskite for Fluorescence Thermometry with Record High Time-Resolved Thermal Sensitivity. *Advanced* *Materials* **35**, 2305495 (2023).
2. Oomen, E., Smit W. & Blasse, G. The luminescence of Cs_2_NaSbCl_6_ and Cs_2_NaSbBr_6_: a transition from a localized to a delocalized excited state. *Chemical Physics Letters* **138**, 23–28 (1987).
3. Zhou, B. et al. Emission Mechanism of Self-Trapped Excitons in Sb^3+^-Doped All-Inorganic Metal-Halide Perovskites. *The Journal of Physical Chemistry Letters* **13**, 9140–9147 (2022).
4. Su, B. B. et al. Highly Distorted Antimony(III) Chloride [Sb_2_Cl_8_]^2-^ Dimers for Near-Infrared Luminescence up to 1070 nm. *Angewandte Chemie International Edition* **134**, e202208881 (2022).
5. Zhou, B. et al. Efficient White Photoluminescence from Self-Trapped Excitons in Sb^3+^/Bi^3+^-Codoped Cs_2_NaInCl_6_ Double Perovskites with Tunable Dual-Emission. *ACS Energy Letters* **6**, 3343–3351 (2021).
6. Jiang, T. M. et al. Highly Efficient and Tunable Emission of Lead-Free Manganese Halides toward White Light-Emitting Diode and X-Ray Scintillation Applications. *Advanced Functional Materials* **31**, 2009973 (2021).
7. Yuan, J. W. et al. Highly Efficient Stable Luminescent Radical-Based X-ray Scintillator. *Journal of the American Chemical Society* **145**, 27095–27102 (2023).
8. Liang, Y. et al. A High-Rigidity Organic-Inorganic Metal Halide Hybrid Enabling Reversible and Enhanced Self-Trapped Exciton Emission under High Pressure. *Nano Letters* **23**, 7599–7606 (2023).
9. Jin, J. C. et al. Rigid Phase Formation and Sb^3+^ Doping of Tin (IV) Halide Hybrids toward Photoluminescence Enhancement and Tuning for Anti-Counterfeiting and Information Encryption. *Angewandte Chemie International Edition* **63**, e202408653 (2024).
10. Liang, D. H. et al. High quantum yield of In-based halide perovskites for white light emission and flexible x-ray scintillators. *EcoMat* **5**, e12296 (2023).
11. Zhou, B. et al. Self-Trapped Exciton Emission in Highly Polar 0D Hybrid Ammonium/Hydronium-Based Perovskites Triggered by Antimony Doping. *Journal of the American Chemical Society* **146**, 15198–15208 (2024).
12. Jing, Y. Y. et al. Sb^3+^ Dopant and Halogen Substitution Triggered Highly Efficient and Tunable Emission in Lead-Free Metal Halide Single Crystals. *Chemistry of Materials* **32**, 5327–5334 (2020).
13. Han, P. G. et al. All-Inorganic Lead-Free 0D Perovskites by a Doping Strategy to Achieve a PLQY Boost from <2% to 90%. *Angewandte Chemie International Edition* **59**, 12709–12713 (2020).
14. Guo, X. X. et al. All-Inorganic Cs_2_YbCl_5_·H_2_O Perovskite with Luminescence Response to Methanol for Anti-Counterfeiting. *Advanced Optical Materials* **12**, 2400681 (2024). .
15. Su, B. B. et al. Sb^3+^-Doping in Cesium Zinc Halides Single Crystals Enabling High-Efficiency Near-Infrared Emission. *Advanced Functional Materials* **31**, 2105316 (2021).
16. Cao, M. Y. et al. Achieving Ultrahigh Efficiency Vacancy-Ordered Double Perovskite Microcrystals via Ionic Liquids. *Small* **18**, 2204198 (2022).
17. Chen, B. et al. Multiexcitonic Emission in Zero-Dimensional Cs_2_ZrCl_6_:Sb^3+^ Perovskite Crystals. *Journal of the American Chemical Society* **143**, 17599–17606 (2021).
18. Cao, L. Y. et al. Strong Self-Trapped Exciton Emission and Highly Efficient Near-Infrared luminescence in Sb^3+^-Yb^3+^ Co-doped Cs_2_AgInCl_6_ Double Perovskite. *Advanced Functional Materials* **33**, 2212135 (2023).
19. Chang, T. et al. Component Engineering to Tailor the Structure and Optical Properties of Sb-Doped Indium-Based Halides. *Inorganic Chemistry* **61**, 1486–1494 (2022).
20. Zhou, B. et al. Efficient White Photoluminescence from Self-Trapped Excitons in Sb^3+^/Bi^3+^-Codoped Cs_2_NaInCl_6_ Double Perovskites with Tunable Dual-Emission. *ACS Energy Letters* **6**, 3343–3351 (2021).
21. Wang, Y. S. et al. Multimode Luminescence Tailoring and Improvement of Cs_2_NaHoCl_6_ Cryolite Crystals via Sb^3+^/Yb^3+^ Alloying for Versatile Photoelectric Applications. *Angewandte Chemie International Edition* **62**, e202311699 (2023).
22. Wang, Z. Y. et al. Boosting the Self-Trapped Exciton Emission in Cs_2_NaYCl_6_ Double Perovskite Single Crystals and Nanocrystals. *The Journal of Physical Chemistry Letters* **13**, 8613–8619 (2022).
23. Chen, C. H. et al. Blue-red dual color emitting phosphor Cs_2_NaLuCl_6_: Sb^3+^, Ho^3+^ for plant growth LEDs. *Ceramics International* **49**, 25232–25239 (2023).
24. Ge, S. G. et al. Realizing Color-Tunable and Time-Dependent Ultralong Afterglow Emission in Antimony-Doped CsCdCl_3_ Metal Halide for Advanced Anti-Counterfeiting and Information Encryption. *Advanced Optical Materials* **11**, 2300323 (2023).
25. Dai, G. K., Ma, Z. M., Qiu, Y. X. & Ma, Z. Y. Codoped 2D All-Inorganic Halide Perovskite Cs_3_Cd_2_Cl_7_:Sb^3+^:Mn^2+^ with Ultralong Afterglow. *Inorganic Chemistry* **62**, 7906–7913 (2023).
